# Effects of a Digital Parent–Child Single-Session Growth Mindset Intervention on Adolescent Depression and Anxiety Symptoms: A Three-Arm Waitlist Randomized Controlled Trial

**DOI:** 10.3390/ejihpe16060084

**Published:** 2026-06-17

**Authors:** Shimin Zhu, Yuxi Hu, Di Qi, An Xi, Shiyun Chen, Ruobing Wang, Paul Lee, Paul Wai Ching Wong

**Affiliations:** 1 Department of Applied Social Sciences, The Hong Kong Polytechnic University, Hong Kong SAR, China; starryyy.hu@connect.polyu.hk (Y.H.); di.qi@yccece.edu.hk (D.Q.); jamie.xi@connect.polyu.hk (A.X.); shiyun.chen@connect.polyu.hk (S.C.); 2 Mental Health Research Centre, The Hong Kong Polytechnic University, Hong Kong SAR, China; 3 Yew Chung College of Early Childhood Education, Hong Kong SAR, China; 4 School of Product Design, University of Canterbury, Christchurch, Canterbury 8041, New Zealand; ruobing.wang@pg.canterbury.ac.nz; 5 School of Pharmacy, The Chinese University of Hong Kong, Hong Kong SAR, China; paullee@pharmacy.cuhk.edu.hk; 6 Department of Social Work and Social Administration, The University of Hong Kong, Hong Kong SAR, China; paulw@hku.hk

**Keywords:** growth mindset, mental health, secondary school students, brief intervention, randomized controlled trial, parent–child interventions

## Abstract

Adolescent depression and anxiety are prevalent, yet brief and scalable parent–child digital interventions remain understudied. This study evaluated the effects of a digital parent–child single-session growth mindset intervention targeting beliefs about intelligence, failure, and emotion on adolescent internalizing of symptoms. In a three-arm waitlist cluster randomized controlled trial, 390 parent–child dyads from seven secondary schools in Hong Kong were assigned to a parent–child intervention group, a child-only intervention group, or a waitlist control group. Students were assessed at baseline, 2 weeks, and 3 months, and parents at baseline and 3 months. Cluster-adjusted generalized estimating equations were used for intention-to-treat analyses. A significant time-by-group interaction was observed for child-reported depression, whereas the interaction effects for other outcomes were non-significant. However, cluster-adjusted baseline differences across groups limited attribution of changes in depression to intervention effects. Relative to controls, the parent–child intervention descriptively showed short-term improvement in hopelessness and sustained gains in child-reported parent–child relationships over 3 months. Moderation analyses suggested clearer short-term benefits among adolescents with higher baseline symptoms and among girls. Overall, the PC-SMILE intervention in this study did not show statistically significant effect on reducing internalizing symptoms. Improvement on intervention design and implementation would benefit further refinement of brief, scalable parent–child digital interventions. Trial registration: ClinicalTrials.gov NCT05493865.

## 1. Introduction

Depression and anxiety disorders are the primary contributors to disability and illness among adolescents globally (World Health Organization, 2020). Globally, 34% of adolescents aged 10–19 are at risk of developing depression (Shorey et al., 2022), while the 12-month pooled prevalence of anxiety disorders is estimated at 9.0% (Biswas et al., 2020). Despite the high prevalence of mental health needs, the majority of adolescents with mental disorders do not receive the help they need. In many low- and middle-income countries, the number of child mental health professionals is extremely limited, often fewer than 0.1 child psychiatrists per 100,000 youth, over 100 times fewer than in high-income countries (United Nations Children’s Fund & World Health Organization, 2022). In Hong Kong, nearly one in four secondary school students report moderate to severe symptoms of depression or anxiety, with approximately one in twelve meeting criteria for a severe disorder (Leung et al., 2008; Mak, 2013). Yet 65% are unable to access mental health services because of long waiting times for public services, high costs of clinic-based care, or fears of stigma and discrimination (Bauhinia Foundation Research Centre, 2017). Experiencing these disorders early in life can heighten the risk of suicide among young people and cause various psychological and occupational difficulties with lasting negative consequences, particularly if not properly treated or supported (Yap et al., 2018). It is therefore important to implement accessible and effective interventions that are family-friendly, affordable, and non-stigmatizing, to provide early support and prevent these conditions from worsening.

Parents play a significant role in either increasing or protecting against adolescent anxiety and depression (Cairns et al., 2014; Yuen et al., 2019). First, both parental and children’s attitudes and coping matter in the face of adversity (Morris et al., 2021), such as structural stress factors like academic pressure (Yuen et al., 2019). A growing amount of research emphasizes the significance of parents’ and children’s growth mindsets (Fuengfoo et al., 2024; Rowe & Leech, 2019). Studies have shown that holding a fixed mindset—the belief that personal attributes cannot be changed—is a significant predictor of mental health issues in young people (Hong et al., 1999; Kneeland et al., 2016; Mueller et al., 2017; Mullarkey & Schleider, 2020; Schroder et al., 2019). Conversely, interventions that encourage a growth mindset—the belief that attributes can be developed—have been found to effectively reduce symptoms of depression and anxiety in adolescents (Schleider & Weisz, 2016, 2018a). Additionally, when parents maintain fixed mindsets about intelligence and emotions, these beliefs create a developmental cascade whereby children internalize similar perspectives about their own capabilities, leading to more internalized problems like depression and anxiety, as well as maladaptive coping with their mental health (Garibaldi et al., 2020; Haimovitz & Dweck, 2016; Schleider et al., 2016; Schleider & Weisz, 2018a, 2018b). Children raised in fixed-mindset environments may develop psychological vulnerabilities characterized by ability doubt, less effective coping mechanisms, and heightened feelings of helplessness (Hong et al., 1999).

As mindset is a modifiable factor (Dweck, 2006), an increasing number of mindset interventions are developed to help adolescents reach their full potential and promote children and youth well-being (Dweck & Yeager, 2019; Yeager & Dweck, 2020). Growth mindset interventions focus on teaching that personal qualities like intelligence, personality, and emotions are changeable, and that improvement is possible through actions such as putting in effort, trying new strategies, learning from mistakes, and seeking help (Schleider et al., 2016; Schleider & Weisz, 2018a; Yeager & Dweck, 2020; Zhu et al., 2025a). These interventions, which typically last between 30 min and an hour, have produced varying effect sizes—from small for academic performance improvement in a large, diverse sample (Yeager et al., 2019) to medium-to-large for anxiety reduction in randomized controlled trials (RCTs; Schleider & Weisz, 2017). Given their short duration and the observed effect sizes across different contexts and populations, brief mindset interventions show strong potential for scalable mental health prevention and early treatment.

The existing growth mindset interventions have primarily targeted growth mindsets in specific areas such as intelligence (Yeager & Dweck, 2020; Yeager et al., 2019), personality (Schleider & Weisz, 2018a, 2018b; Yeager et al., 2014), and emotions (Zhu et al., 2025a) to enhance academic performance, build resilience, and alleviate mental health symptoms. These interventions typically delivered single-domain content in digital formats with relatively short session lengths (often 20–60 min), and samples of parents and adolescents from school settings were enrolled. While these domain-specific approaches have shown positive results, no intervention has yet combined multiple core growth mindsets into an integrated mindset program. Yeager and colleagues have found a synergistic effect of integrating stress mindset and growth mindset for stress management (Yeager et al., 2022; Yeager & Dweck, 2020). This finding is particularly relevant given that the causes of youth depression and anxiety are multifaceted—involving academic pressure, life setbacks, uncertainty, and emotional challenges during adolescence—and may therefore require comprehensive intervention approaches. This study seeks to fill this gap by developing and evaluating an integrated intervention that targets multiple growth mindsets to address internalizing symptoms in youth.

Parental mindsets significantly influence adolescents’ own mindsets and their risk for internalizing problems like depression and anxiety (Haimovitz & Dweck, 2016; Schleider & Weisz, 2016). While parents are a primary source of support for teenagers, they can also contribute to stress if they hold a fixed mindset and view failure as harmful. For instance, when parents believe intelligence is unchangeable, their children are more likely to experience symptoms of depression and social anxiety due to increased worries about performance and fear of negative judgment (Schleider et al., 2016). Additionally, parents’ attitudes toward failure shape how they respond to their children’s setbacks (Haimovitz & Dweck, 2016). Parents who see emotions as fixed and believe failure is damaging are more likely to prefer passive approaches to addressing their children’s mental health concerns (Schleider & Weisz, 2018b). On the other hand, fostering beliefs in the potential for change regarding intelligence and emotions can help reduce family anxiety about failure and mental health issues, lessen stigma, boost feelings of control, encourage proactive help-seeking, and improve parent–child relationships. When families view abilities as developable rather than fixed, they are more likely to interpret setbacks as temporary challenges that can be overcome through effort, rather than as permanent deficits that define a child’s capabilities. Given the important role parents play in preventing internalizing problems in children (Yap et al., 2016), joint growth mindset interventions for both parents and children hold promise for enhancing the well-being of families.

This study aims to address three key gaps. First, most existing interventions target fixed mindsets in only one area—such as intelligence, personality, or emotions (Schleider & Weisz, 2018a; Zhu et al., 2025a)—and rarely combine multiple relevant mindset domains closely linked to youth mental health within a single program. Integrating several relevant mindset domains into one intervention could enhance its effectiveness. Second, we aimed to develop a parent–child mindset intervention in the Chinese context. Given that Chinese parents often experience high levels of anxiety about their children’s education (Xinhua Net, 2018) and that parental mindsets may have an even greater impact on children’s mental health (Fong, 2007), a parent–child mindset intervention would address this significant need. To rigorously assess the value of parental involvement, this study employs a child-only comparator that uses the same core child-focused content, instructional length, and delivery modality as the parent–child condition but excludes parental engagement. Third, despite strong evidence supporting the benefits of preventive parenting programs, these interventions are often underutilized due to obstacles like scheduling conflicts and concerns about privacy (Yap et al., 2018). It is thus crucial to develop brief, easily accessible, and non-stigmatizing interventions for both parents and children.

This study developed and evaluated an online parent and child single-session intervention on mindsets of intelligence, failure, and emotion (PC-SMILE; Zhu et al., 2024). We used a web-based intervention as it is found to be promising to address scaling mental health service needs in the digital era (Löchner et al., 2025). Implementing the single-session web-based integrated mindset intervention is an initiative to provide a scalable and accessible intervention that can benefit children and parents. We aimed to examine the efficacy of the intervention among first-year secondary school students (equivalent to Grade 7 in the American education system) as they are navigating increased academic and social challenges during a transitional period to middle school, while their parents are remaining highly engaged and collaborative in a parent–child intervention program. The current study examined the efficacy of PC-SMILE for adolescents and their parents, compared to a child single-session intervention on mindsets of intelligence, failure, and emotion (C-SMILE) group and a waitlist control group. Using C-SMILE as a comparison group allows us to examine whether the efficacy of a joint parent–child intervention is better than that of a child-only intervention and a waitlist control.

### 1.1. Objectives

The primary objective was to test the efficacy of PC-SMILE in reducing depression and anxiety symptoms in secondary school students. The secondary objective was to evaluate the efficacy of PC-SMILE in (a) reducing hopelessness and perceived parent performance (vs. learning) orientation and enhancing mental well-being and academic self-efficacy, and (b) enhancing parent–child interactions and relationships. Both students and parents completed validated measures. Student outcomes were assessed in the student survey at baseline, and 2-week and 3-month follow-ups, while parental outcomes (e.g., parents’ perceived children’s depression and anxiety symptoms) were measured in the parent survey at baseline and 3-month follow-up.

### 1.2. Hypotheses

**H1.**  *The PC-SMILE group is more effective in the primary outcome (i.e., reducing anxiety and depression symptoms in student participants) than both the C-SMILE group and the waitlist control group at the 2-week and 3-month follow-ups.*

**H2.**  *The PC-SMILE group is more effective in the secondary outcomes of (i) reducing hopelessness, (ii) enhancing mental well-being, (iii) enhancing academic self-efficacy, (iv) reducing perceived parent performance (vs. learning) orientation, and (v) enhancing parent–child relationships than both the C-SMILE group and the waitlist control group at the 2-week and 3-month follow-ups.*

## 2. Materials and Methods

### 2.1. Ethical Considerations

Ethical approval was granted by the Institutional Review Board of the Hong Kong Polytechnic University (reference number: HSEARS20211009001). Participation was voluntary. Informed written consent was obtained from all participating parents and students. Participants were informed of their right to withdraw from the intervention or to decline to answer any questions on the questionnaire, and they were assured that their responses would remain confidential. Additional information for referral services was provided to participants with clinical-level mental health needs. The data was de-identified (for example, by removing school names and assigning unique codes) and kept in secure storage. Participants’ personal data will not be disclosed in any publication or reporting.

### 2.2. Study Design

**Figure 1:**
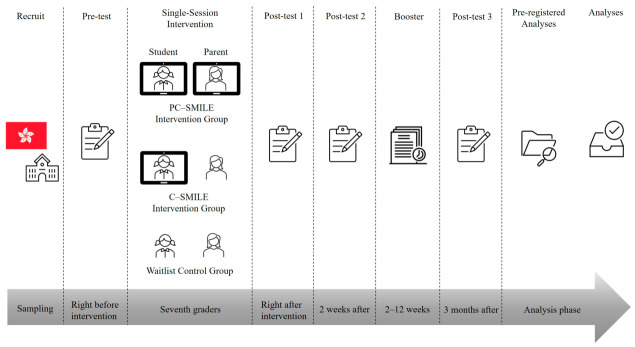
Design of the three-arm waitlist randomized controlled trial.

This study was a three-arm cluster-RCT of adolescents and their parents in Hong Kong (Figure 1). A study protocol was previously published (Zhu et al., 2024). The trial was registered at ClinicalTrials.gov (NCT05493865).

Cluster randomization was conducted at the class level. Classes in each eligible school were randomized (using computer-generated random numbers) into the PC-SMILE, C-SMILE, or waitlist control group. For the PC-SMILE group, both students and parents received the intervention, while for the C-SMILE group, only students received the intervention. The waitlist group was invited for the PC-SMILE intervention after the three-month follow-up survey. All student participants received regular educational activities and interventions in school. Three repeated assessments of the measures were conducted simultaneously for the children in the three groups at (i) baseline, (ii) two weeks post-intervention, and (iii) three months post-intervention. Parents in the three groups were measured at (i) baseline and (ii) three months post-intervention.

### 2.3. Participants

Eligible adolescents in Grade 7 and their parents were recruited from seven secondary schools. The inclusion criteria for adolescent participants included (1) aged between 11 and 16 with one parent willing to participate, (2) Chinese youth who can read and write Chinese, (3) sufficient visual and auditory abilities to complete the intervention and assessment and (4) able to give consent to participate in the study. The exclusion criteria for adolescent participants included (1) no parental consent; (2) unable to remain focused on completing the intervention and the survey, which took approximately 45 and 25 min, respectively; and (3) having intellectual disability or severe illness or pain that could introduce significant bias in the students’ health and mental health situation.

The inclusion criteria for parent participants were (1) living with the child participant, (2) Chinese-language proficiency and comfort with completing computer-based activities, (3) sufficient visual and auditory abilities to complete the intervention and assessment, and (4) ownership of personal internet-equipped devices. The exclusion criteria for parent participants were (1) having mental illnesses that may introduce significant bias in the survey responses, as well as exclusion criteria (2) from the above criteria for adolescent participants.

For students, exclusions were determined via teacher report and student self-report at baseline. For parents, exclusions were determined via parent self-report in the survey (with clarification when needed).

### 2.4. Procedure

The school and student recruitment process included the following two steps. First, study invitations were sent to schools chosen randomly from the secondary school list across Hong Kong Island, Kowloon, and the New Territories. Invitations stopped when seven schools had agreed to participate, as this was expected to be sufficient to achieve the target sample size. However, the final sample size was slightly below the original target, as school scheduling constraints prevented further recruitment. Second, within each participating school, three classes in Grade 7 were selected using random numbers, and all students in these classes were invited to join the study. Randomization was conducted at the class level rather than the individual level: each selected class, as a whole, was assigned by computer-generated random numbers to the PC-SMILE group, the C-SMILE group, or the waitlist control group.

Students and parents completed all study procedures via personal internet-equipped devices. They received the link of research material and completed the Qualtrics-based baseline survey, intervention, and follow-up surveys independently. For student participants, the interventions were conducted in school classrooms or activity rooms which were equipped with sufficient computers or tablets, and headphones. All intervention activities were self-administered and delivered in a web-based format. The research team and trained research assistants remained in the intervention rooms to provide help if needed. Parent participants received a link to the baseline Qualtrics survey and the intervention (for the PC-SMILE group). The interventions for parents and students both lasted around 45 min but were different in terms of narrative and content. Between the 2-week and the 3-month follow-up survey, a total of 5 weekly booster messages with core intervention contents were sent to the intervention groups every 2 weeks. After completing the 3-month follow-up survey, parents in the C-SMILE or the waitlist control group who did not receive the intervention previously would receive the intervention link.

Each participating family received a HK$100 (≈US$12) supermarket voucher after the baseline assessment and the 3-month follow-up, respectively, and the total compensation for each family was HK$200 (≈US$24). The compensation amount and schedule were identical across all three groups and were tied to each assessment completion rather than intervention assignment or participants’ responses. The compensation was intended to facilitate participation and retention and was unlikely to differentially influence study outcomes.

Surveys across time points and within parent–child dyads were linked using a unique code constructed from the school code, grade, class, and the student’s roll number within the class. At the beginning of each survey, both students and parents entered the same information, which allowed us to generate code and match each participant’s baseline, 2-week, and 3-month surveys, as well as linking each parent with their child in the dataset. Records with inconsistent or missing codes were reviewed and corrected when possible before analysis.

### 2.5. Trial Power and Sample Size

A small effect size (Cohen’s *d* = 0.33) was assumed based on previous research (Schleider & Weisz, 2018a). With a power of 0.80 and an alpha of 0.05, a sample size of 438 dyads (146 per arm) was required to ensure that the sample size was sufficient to test the hypotheses. As the attrition rate from our previous study was very low (Zhu et al., 2025a), the baseline recruitment target was set at 450 participants (150 per arm). After excluding students who were absent or withdrawn from school and those who did not complete the intervention, we finished with 390 participants.

### 2.6. Measures

Participants were guided to read the instructions of the survey and complete the self-rated scales, which took approximately 25 min.

#### 2.6.1. Content Fidelity Checking for Both Parent and Student Participants

Mindset about intelligence was measured using the three-item Implicit Theory of Intelligence Scale, which assesses parents’ and children’s beliefs about the malleability of intelligence (Dweck et al., 1995; Zhu et al., 2020). A sample item includes ‘You have a certain amount of intelligence and there is really not much you can do to change it’. Each item was rated on a 6-point scale ranging from 1 (strongly disagree) to 6 (strongly agree). A higher mean score of the three items indicated a lower belief-in-change (a more fixed mindset) in one’s intelligence. The Cronbach’s *α* of the scale in the current study was 0.85 for children and 0.90 for parents at baseline, showing good reliability.

Mindset about failure was measured by the six-item Implicit Theory of Failure Scale (Haimovitz & Dweck, 2016), which evaluates parents’ and children’s views of failure as enhancing or debilitating on a 6-point Likert scale ranging from 1 (strongly disagree) to 6 (strongly agree). An example item of an enhancing belief is ‘The effects of failure are positive and should be utilized’, and an example item of a debilitating belief is ‘The effects of failure are negative and should be avoided’. Enhancing belief items were reverse-scored and a higher mean score of the six items indicated more debilitating beliefs. The Cronbach’s *α* in the current study was 0.75 for children and 0.76 for parents at baseline.

Mindset about emotion was assessed using the validated Chinese version of the 12-item Mindset of Depression, Anxiety, and Stress Scale (MDASS; Zhu et al., 2022) to measure children’s belief in the changeability of negative emotion states. Sample items include ‘When you have a certain level of depression/anxiety/stress, you really cannot do much to change it’. The Cronbach’s *α* of the scale in the current study was 0.96 at baseline. Parents’ mindset about emotion was measured by a 4-item adapted version of MDASS, which includes items such as ‘If they want to, people can change the emotions they have’. Each item was rated on a 6-point Likert scale ranging from 1 (strongly disagree) to 6 (strongly agree), and a higher mean score indicated a more fixed mindset regarding the changeability of negative emotional states. The Cronbach’s *α* was 0.69 at baseline.

#### 2.6.2. Primary Outcome

Children’s depression and anxiety symptoms were measured using the 25-item Revised Children’s Anxiety and Depression Scale (RCADS-25) among both children and parents (Ebesutani et al., 2012, 2017). Sample items measuring anxiety and depression include ‘I/My child worry when I/My child think I/My child have done poorly at something’ and ‘I/My child feel sad or empty’. Each item was rated on a 4-point Likert scale ranging from 0 (never) to 3 (always), indicating the frequency over the past two weeks. The child survey has an overall score (range: 0–75), a total anxiety score from the anxiety subscale (range: 0–45; Cronbach’s *α* = 0.88 at baseline), and a total depression score from the depression subscale (range: 0–30; Cronbach’s *α* = 0.89 at baseline).

#### 2.6.3. Secondary Outcomes

Hopelessness was measured by the four-item Helplessness subscale of the Demoralization Scale (Kissane et al., 2004) among the child participants. Each item was scored on a 5-point Likert scale ranging from 1 (strongly disagree) to 5 (strongly agree), with a higher mean score of the four items indicating a greater level of hopelessness. An example item is ‘I feel hopeless.’ The Cronbach’s *α* of the Chinese version of this Helplessness subscale was 0.91 at baseline in the current study.

Mental well-being was measured by the short Warwick-Edinburgh Mental Well-Being Scale (Ng et al., 2014; Stewart-Brown & Janmohamed, 2008) among the child and parent participants. It contains seven items rated on a 5-point Likert scale ranging from 1 (none of the time) to 5 (all the time), with a higher total score of the seven items indicating a higher level of participants’ mental well-being. A sample item is ‘I have been feeling optimistic about the future.’ The Cronbach’s *α* was 0.89 for children and 0.88 for parents at baseline.

Parent–child interactions were measured using three self-developed questions regarding the number of days per week that children spend more than 15 min per day engaging in activities with their parents. Children were asked to rate the frequency (the number of days) on an 8-point scale ranging from 0 (None) to 7 (seven days). The three items were about parenting activities, including chatting, hanging out, and watching TV/movies/videos. A higher mean score of the three items indicated greater parent–child interactions. The Cronbach’s *α* was 0.71 at baseline.

Parent–child relationships were measured using three self-developed items in both children and parents. For children, the three items were ‘I am very satisfied with the relationship between me and my parents,’ ‘I proactively tell my family what had happened to me,’ and ‘I proactively share my feelings with my family.’ The adapted items ‘My child is very satisfied with the relationship between him/her and us,’ ‘My child proactively tells us what has happened to him/her,’ and ‘My child proactively shares his/her feelings with us’ were used for parents. Each item was rated on a 4-point Likert scale ranging from 1 (strongly disagree) to 4 (strongly agree) and a higher mean score indicated a better parent–child relationship. The Cronbach’s *α* was 0.87 for children and 0.86 for parents at baseline.

Academic self-efficacy was measured using an adapted five-item scale as part of the Patterns of Adaptive Learning Survey (Friedel et al., 2007). Children were asked to rate the extent to which they anticipated the skills taught in class could be mastered and the work could be done on a 6-point Likert scale ranging from 1 (strongly disagree) to 6 (strongly agree). A sample item is ‘I’m certain I can master the skills taught in class.’ A higher mean score of the five items indicated a higher level of academic self-efficacy. The Cronbach’s *α* was 0.91 at baseline.

Perceived parent learning versus performance orientation were assessed in children with the eight items used by Haimovitz and Dweck (2016). Four items measured children’s perceptions of their parents’ performance orientation, and the other four measured their perceptions of their parents’ learning orientation. The scale asked children about the parents’ responses to a scenario when they got a poor grade on a quiz. A sample item of performance-orientation is ‘My parents don’t like it when I make mistakes in school’ and a sample item of learning-orientation is ‘My parents want me to understand school concepts, not just do the work.’ All the items were rated on a 6-point Likert scale ranging from 1 (strongly disagree) to 6 (strongly agree). The learning-focused items were reverse-scored, and then responses to all items were averaged, with a higher mean score of the eight items indicating more agreement with a performance (vs. learning) orientation of parents in children’s perceptions. The Cronbach’s *α* in the current study was 0.75 for perceived parent learning orientation and 0.70 for perceived parent performance orientation at baseline.

The motivation for applying the contents learned from the intervention was assessed right after the intervention. Child participants were asked to rate the extent to which they would like to apply what was learned from the intervention to address future challenges and the extent to which they would like to improve their ability to cope with challenges. Parent participants were asked to rate the extent to which they would like to apply what was learned from the intervention to help their child to address future challenges and the extent to which they would like to improve the way they respond to their child. All motivation items were rated on a 6-point Likert scale ranging from 1 (not very likely) to 6 (very likely).

The intervention feedback scale was used to assess the acceptability of parent and child interventions. The scale asked children and parents to indicate the extent to which they liked, understood, felt helped by, and would recommend the program, as well as the extent to which they found the program interesting and agreed with its message (Schleider et al., 2020) on a 5-point scale (such as from 1 = strongly disagree to 5 = strongly agree). They were also prompted to provide written feedback about this program.

Attention-checking items were included to check participants’ attention and ensure data quality. Two attention-checking items at all assessment points (baseline and follow-up surveys) and one item at the post-intervention survey for fidelity checking and intervention feedback were included for child participants. Attention-checking items were also included in the parent surveys: two items at baseline, one at post-intervention, and one at the three-month follow-up survey. These items asked participants to follow the instructions to select a specific option. A sample item is “Please select ‘strongly agree’ for this item.”

Socio-demographic information was collected, covering a range of characteristics of child participants (gender, age, ethnicity, if an only child, and if living with parents) and parent participants (relation with child, ethnicity, age range, marital status, education level, employment status, and household monthly income) at baseline to examine variability between groups.

### 2.7. Data Analysis

An intention-to-treat analysis was used as the primary analysis, with all randomized participants analyzed according to their originally assigned groups. Participants who completed all assessments and passed all attention-checking items at every assessment point were classified as the per-protocol population. A per-protocol analysis was performed as a sensitivity analysis. As the cluster unit is class, the effect of the cluster randomization of classes within the same school was accounted for (Jo et al., 2008). One-way ANOVA was first conducted to examine baseline group differences overall and within each school. Two-way ANOVA further examined the group-by-school interaction effects. School was only included in generalized estimating equation (GEE) models when significant group-by-school interaction effects were observed. GEE analyses at the class level were used to examine the group effect, time effect, and group-by-time interaction effect on outcome measures (Liang & Zeger, 1986; M. Wang, 2014). A statistically significant interaction effect signified the effectiveness of the interventions. This cluster-adjusted approach appropriately handled the intraclass correlation resulting from class-level randomization. In addition, the effect sizes using estimated marginal means were calculated. These effect sizes (Cohen’s *d*) compared mean gain scores reflecting changes in each outcome from baseline to the two follow-ups for child participants receiving the interventions vs. waitlist controls. The effect sizes were also compared between participants in the PC-SMILE and those in the C-SMILE intervention group. For outcomes measured in parents, GEE analyses and effect size calculations were also conducted to examine the intervention effects, following the same procedure as above. Furthermore, for exploration, we tested the following moderators on the effectiveness of the interventions for the outcome measures: children’s depression and anxiety levels at baseline (high vs. low, dichotomized by the median of the baseline RCADS-25 total score) and gender (boys vs. girls). The corrected quasi-likelihood under the independence model criterion (QICC) values for the models with and without the moderator were compared, and a smaller QICC for the models including the moderator indicated evidence of a moderation effect. Multiple comparisons were not conducted, and a *p* value < 0.05 indicated statistical significance. SPSS (version 26; IBM Corp, Armonk, NY, USA) was used for all statistical analysis.

## 3. Results

### 3.1. Recruitment and Randomization

**Figure 2:**
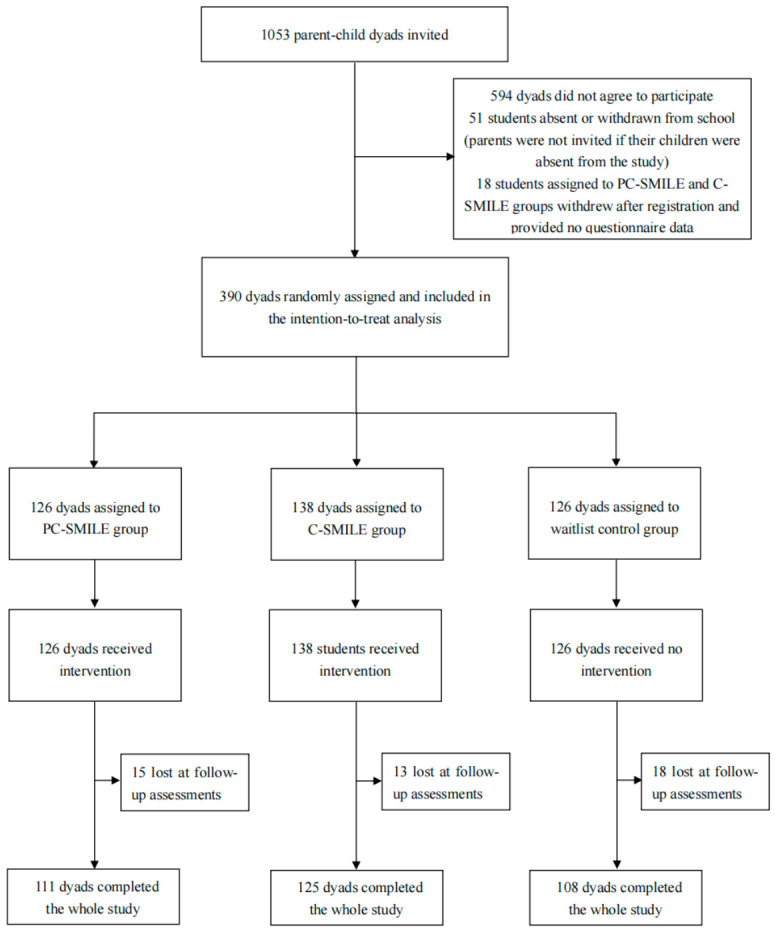
CONSORT flowchart.

The CONSORT diagram of the recruitment and participation flow is depicted in Figure 2. A total of 390 dyads were recruited and randomized into three groups, namely PC-SMILE (126, 32.3%), C-SMILE (138, 35.4%), and waitlist control (126, 32.3%). All parent–child dyads in the PC-SMILE group and children in the C-SMILE group received the interventions. Child participants were contacted for both the 2-week and 3-month follow-ups, and parent participants were followed-up at 3-month post-intervention. The proportions lost to follow-up were 11.9% in the PC-SMILE group, 9.4% in the C-SMILE group, and 14.3% in the waitlist control group. The primary analysis followed the intention-to-treat principle. However, 18 participants who withdrew after registration and provided no questionnaire data at any time point could not be included in the GEE outcome analyses.

### 3.2. Participant Characteristics at Baseline

The baseline characteristics of the 390 dyad participants are summarized in Table 1. A total of 57.2% of the children were girls (n = 223). Only statistical differences in children’s age were found among groups. There were no statistical differences in other demographic characteristics of child participants or in any characteristics of parent participants. Additionally, before class-level cluster-adjustment, there were no significant differences among groups in children’s mindsets, primary and secondary outcomes, or in parental mindsets and outcome measures (see Supplementary Figures S1–S4).

### 3.3. Fidelity Checking of Mindsets

Fidelity checking results of mindsets right after intervention compared to baseline showed that students’ mindsets of intelligence (*p* = 0.004 for PC-SMILE and *p* < 0.001 for C-SMILE) and mindsets of emotion (*p* = 0.003 for PC-SMILE and *p* = 0.001 for C-SMILE) significantly changed to more growth after intervention; their mindsets of failure also changed to be more growth-oriented though not reaching significance (*p* = 0.051 for PC-SMILE and *p* = 0.11 for C-SMILE). For parental mindsets, their mindsets of intelligence (*p* < 0.001) and mindsets of failure (*p* < 0.001) significantly changed to more growth after intervention (i.e., PC-SMILE), while mindsets of failure tended to more growth as well though not reaching significance (*p* = 0.13).

### 3.4. Changes in Primary and Secondary Outcomes of Children

For the primary outcome of children’s depression and anxiety symptoms measured by RCADS-25 (child version), we found no significant main effects of group for the overall (*p* = 0.33), anxiety (*p* = 0.46), and depression (*p* = 0.23), and significant main effects of time for the overall (*p* = 0.004) and anxiety (*p* < 0.001) with non-significance in depression (*p* = 0.10). The time-by-group interaction effects were non-significant for the overall RCADS scores (*p* = 0.72) and anxiety symptoms (*p* = 0.53), but significant for depressive symptoms (*p* < 0.001), indicating that symptom trajectories over time differed across groups only for depression (see Table 2). However, the clustered-adjusted GEE results showed substantial between-group differences among three groups at baseline for depressive symptoms (i.e., all between-group differences among PC-SMILE, C-SMILE, and waitlist control group were statistically significant at baseline, all *P*s < 0.001). Thus, the post-intervention between-group differences in depressive symptoms cannot simply be attributed to the intervention itself, as they may also reflect baseline differences. Notably, before clustered-adjustment, Table 1 showed that observed baseline means did not indicate significant between-group differences, whereas the cluster-adjusted GEE estimated marginal means accounting for both school- and class-level clustering suggested baseline differences across groups for RCADS depression and several secondary outcomes. This suggests that the estimated between-group differences may be sensitive to the clustering structure used in the model. School- and class-level clustering may also influence intervention effects.

Within-group time contrasts in the PC-SMILE group suggested a short-term improvement in internalizing symptoms at the 2-week follow-up, followed by partial relapses at the 3-month follow-up. When comparing the changes from baseline to follow-ups between each pair of groups, the effect sizes ranged from very small to small (see Table 3). Taken together, the significant interaction effect for depression and the within-group patterns among the three groups may reflect differential change over time. However, the presence of baseline differences for depression, together with the absence of interaction effects for the overall RCADS-25 score and anxiety, suggests that these results should be interpreted cautiously and do not provide robust evidence of superior intervention efficacy of PC-SMILE relative to the other two control groups.

For the secondary outcomes, no significant time-by-group interaction effects were observed for hopelessness (*p* = 0.31), mental well-being (*p* = 0.06), parent–child interactions (*p* = 0.50), parent–child relationships (*p* = 0.20), academic self-efficacy (*p* = 0.49), or perceived parent performance (vs. learning) orientation (*p* = 0.58; see Table 2). These findings indicated that there was no robust evidence of differential change over time across groups for the child secondary outcomes.

Among the secondary outcomes, improvement on hopelessness and parent–child relationships was observed in the PC-SMILE group. Specifically, within-group contrasts suggested a short-term improvement in hopelessness at the 2-week follow-up (*p* = 0.031). Additionally, parent–child relationships improved from baseline to both follow-up assessments and appeared to show a relatively sustained pattern over time (*p* = 0.005 at the 2-week follow-up; *p* = 0.002 at the 3-month follow-up). The corresponding effect sizes for these outcomes were very small to small (see Table 3). However, no significant time-by-group interaction effects were found and the improvement should be interpreted cautiously, indicating that PC-SMILE is not superior to the C-SMILE or waitlist control group. For the remaining secondary outcomes, given the absence of interaction effects, the between-group differences likewise cannot provide robust evidence on intervention efficacy.

The sensitivity analysis was conducted for the per-protocol population. As no waitlist control participants met the per-protocol criteria, the analysis only included the PC-SMILE and C-SMILE groups. Findings showed that significant time-by-group interaction effects only existed for academic self-efficacy (*p* = 0.042), whereas the interaction effects were non-significant for the primary outcomes and all other secondary outcomes. For academic self-efficacy, however, substantial between-group differences were already present at baseline (*p* = 0.006); therefore a clear comparison of intervention-related change between the two groups was not possible. Overall, the per-protocol analysis was largely consistent with the main analysis in showing limited evidence for robust intervention effects. The specific results of the sensitivity analysis can be seen in Supplemental Table S1.

### 3.5. Changes in Parental Outcomes

Parents’ perceived children’s depression and anxiety symptoms measured by the RCADS-25 parent version significantly increased from baseline to the 3-month follow-up (all *P*s < 0.001 for the overall, anxiety, and depression scores). However, the PC-SMILE group exhibited the smallest increase relative to two control groups (see Supplemental Table S2). No significant group effects were found for the overall (*p* = 0.24), anxiety (*p* = 0.22), and depression (*p* = 0.34) scores. The time-by-group interaction effects were non-significant for the overall RCADS score (*p* = 0.21), anxiety (*p* = 0.29), and depression (*p* = 0.22), indicating that there was no statistical evidence of differential change over time across groups.

Regarding parental mental well-being, only the PC-SMILE group showed a significant within-group improvement from baseline to 3 months (*p* = 0.017), whereas the time-by-group interaction was not significant (*p* = 0.26). For parent-reported parent–child relationships, no significant within-group changes were observed in any group, and the interaction effect was also non-significant (*p* = 0.38). The effect sizes for the comparisons between groups on the changes from baseline to the 3-month follow-up were all small (Supplemental Table S3).

### 3.6. Moderation Analysis

Moderation analyses were conducted to examine whether intervention effects differed according to (a) children’s baseline depression and anxiety symptom level (see Supplemental Table S4 for details) and (b) gender (see Supplemental Table S5). For baseline symptom level, children were classified as having relatively high or low symptoms using a median split of the baseline RCADS-25 total score. For gender, the moderation analysis examined whether the intervention effect, indexed by the time-by-group interaction, differed between boys and girls.

Including baseline children’s depression and anxiety symptom level as a moderator improved all model fit except for the outcome of perceived parent performance (vs. learning) orientation, as indicated by the decreases in QICC, ranging from 15.61 to 78,408.07. Most time-by-group interaction effects were non-significant across the high- and low-RCADS subgroups, and for some outcomes baseline between-group differences were evident, which limited further group comparisons. Descriptively, children with higher baseline depression and anxiety symptoms appeared to show greater short-term improvement than children with lower baseline symptoms, particularly for internalizing symptoms. In the high-RCADS subgroup, reductions were more apparent from baseline to the 2-week follow-up in the PC-SMILE and C-SMILE groups, followed in some cases by partial relapses at the 3-month follow-up. These patterns may suggest that children with elevated baseline symptoms were more likely to show short-term benefit, but this interpretation remains descriptive rather than confirmatory.

Including the moderator of gender improved model fit for the outcomes of children’s depression and anxiety symptoms (the overall scores and two subscale scores), well-being, parent–child interactions, and academic self-efficacy. The decreases in QICC ranged from 1.67 to 2519.16. Overall, evidence for gender moderation was also limited, as most time-by-group interaction effects were non-significant or some subgroup comparisons were difficult to interpret because of baseline differences. Descriptively, however, girls appeared to show more favorable changes than boys across several outcomes. For the RCADS-25 overall, anxiety, and depression scores, reductions from baseline to the 2-week follow-up were more consistently observed in girls across the intervention conditions, whereas boys showed fewer statistically significant within-group changes over time. A similar pattern was observed for parent–child relationships, for which improvements in girls were more apparent across both follow-ups in the PC-SMILE group, whereas corresponding changes in boys were only observed at the 3-month follow-up. Mental well-being also showed a similar pattern, with boys exhibiting a significant decline in C-SMILE group (from 25.4 to 23.5; *p* = 0.013) whereas girls in that group showed significant improvements (from 25.4 to 26.2; *p* = 0.008). Taken together, these findings suggest that girls may have benefited more than boys across internalizing symptoms, relational, and well-being outcomes, although this pattern should be interpreted as descriptive rather than confirmatory as well.

An additional exploratory subgroup analysis was conducted among children whose baseline RCADS-25 scores were at or above the borderline clinical threshold for anxiety and depression (T-score ≥ 65), yielding a subgroup of 64 participants (16.4%). Because RCADS-25 clinical thresholds are defined using T-scores, baseline raw scores were first converted to T-scores to identify eligible participants. For Grade 7 children, this threshold corresponded to raw scores of ≥30 for boys and ≥35 for girls (RCADS-25 Manual Scoring, n.d.). Using the same cluster-adjusted GEE approach as in the main analysis, results showed significant baseline between-group differences. These subgroup findings therefore should be interpreted cautiously and did not provide robust evidence that PC-SMILE was superior to the control groups.

### 3.7. Intervention Feedback

Participants’ feedback on the interventions was detailed in Table 4. Around 70% of student participants and around 85% of parent participants indicated general acceptance of this course. Parent participants had more positive feedback than child counterparts on basically all specific feedback items.

## 4. Discussion

The purpose of this study was to examine the efficacy of a web-based single-session parent–child intervention using an integrated mindsets approach (i.e., PC-SMILE) among secondary school students. Using a three-armed RCT, this study compared the efficacy of PC-SMILE with two control groups: child participants only and a waitlist control. Fidelity checking indicated significant immediate changes in mindsets. Overall, the current trial did not find evidence that the PC-SMILE group was superior to the child-only or waitlist control conditions in reducing children’s depression and anxiety symptoms. Although descriptive patterns showed improvements for internalizing symptoms, hopelessness, and parent–child relationships in the PC-SMILE group, the corresponding time-by-group interaction effects were statistically non-significant.

Cluster-adjusted baseline differences exist for depression symptoms, mental well-being, parent–child interactions, and academic efficacy. These differences therefore limited attribution of subsequent between-group differences to the intervention itself. A recent systematic review found that parental involvement in digital cognitive behavioral therapy interventions for children aged 12 and under yielded beneficial effect for reducing anxiety; however, such benefits were not observed in psychoeducation-based programs (Grajdan et al., 2025). The current single-session parent–child program did not identify significant between-group differences in anxiety and depression outcomes. We will discuss the potential, findings, limitations, and insights of this clinical trial for improvements in brief digital mental health intervention design and implementation.

### 4.1. Potential of Integrated Mindset Intervention

Integrated mindset intervention focusing on changing mindset on intelligence, failure and emotion (SMILE) has potential for mental health. This study is the first trial among secondary school students and parents. Although significant intervention effects were not found in this trial, the current findings highlight some potential benefits in the PC-SMILE group. Children in the PC-SMILE group perceived improved internalizing symptoms, parent–child relationships and hopelessness, and parents themselves also reported improved well-being three months after the intervention. Parents with more growth mindsets and failure-is-enhancing mindsets may see setbacks as an opportunity to learn and be more patient and supportive of children (Haimovitz & Dweck, 2016). Similarly, positive effects were observed in other SMILE-series integrated mindset interventions. For example, the We-SMILE intervention eased practicum-related anxiety among social work practicum trainees (Y. Wang et al., 2025). Intervention design tailored for target populations, careful implementation strategies for brief interventions, and suitable timing are crucial to ensure the effectiveness of the SMILE intervention.

### 4.2. Discussion on Parent–Child Intervention-Related Findings

Parent–child interventions can benefit youth mental health. From the within-group perspective of the current trial, child participants in the PC-SMILE group reported improved parent–child relationships, and parents reported higher well-being, with both improvements maintained at the 3-month follow-up. Moreover, PC-SMILE achieved higher retention rates at follow-ups than those in the control groups. As mental health needs among adolescents are increasing (Biswas et al., 2020), joint parent–child interventions focusing on cultivating growth mindsets may simultaneously benefit both parents and children. Interestingly, there are high discrepancies in parent- and child-reported mental health symptoms. Parents across all groups reported worsened child depression and anxiety symptoms at the 3-month follow-up, whereas children reported relatively stable symptoms over the same period. These parent–child informant discrepancies are consistent with prior findings (De Los Reyes et al., 2008; Goolsby et al., 2018). One possible explanation is that parents may hold higher expectations for children’s mental health, leading to lower acceptance and heightened sensitivity to emerging symptoms. Qualitative inquiry would be helpful to examine the potential reasons and mechanisms. Lastly, parents reported higher acceptance and satisfaction with the intervention than children did, suggesting that parents may have higher demands for such interventions to improve parenting and support children.

### 4.3. Discussion on Moderations

Baseline symptoms severity and gender showed mild but consistent moderation effects. Adolescents with higher baseline symptoms appeared to show greater short-term improvement than those with lower symptom levels. This aligns with prior research indicating that youth with greater initial symptom severity benefit more from digital mental health programs, likely because such interventions address their most immediate needs (Zhu et al., 2025b). Meanwhile, girls generally showed more favorable changes than boys across several symptom, relational, and well-being outcomes. The stronger relational effects among girls may reflect established gender differences in emotional openness, help-seeking, and responsiveness to psychosocial and family-focused interventions, with girls typically more engaged in such support (Salk et al., 2017). These findings suggest that brief digital interventions like PC-SMILE may be especially valuable for high-risk groups, including those with elevated internalizing symptoms and females, to maximize the effectiveness of school-based mental health strategies.

### 4.4. Limitations and Suggestions for Future Studies

Some limitations of the current trial should be highlighted to ensure cautious use of the results and to inform future studies. First, class-level cluster design may cause less rigorous results than individual-level RCT (Bolzern et al., 2018; Easter et al., 2021). We assumed minimum class baseline differences based on the school information, and thus we used class randomization to facilitate the intervention process in a school-based setting. However, the baseline differences in class were unpredictable and turned out to exist for some outcomes. Thus, we suggest using individual-level RCT in future studies, especially for interventions with small to medium effect sizes. Second, we cannot observe many of the expected efficacies that the other SMILE-series intervention found (Y. Wang et al., 2025), as the general population we targeted and the timing may have hindered the effect. The moderation analyses of the current study and other studies showed that participants with higher symptoms reported more improvements (Zhu et al., 2025a). The timing of the 3-month follow-up clashed with the examination time which is generally a stressful time for students. As brief digital interventions may show only small to medium effect sizes (Schleider & Weisz, 2017), carefully dedicated design and sensitive timing should be considered to avoid diluting the effects and introducing confounders that may mask the original impact. Third, despite web-based single-session interventions being inherently accessible and scalable, the current experiment setting did not encourage both students and parents to re-access intervention materials, which may have hindered the potential for sustained benefits. Fourth, outcome measures relied on self-reported surveys, which may be susceptible to social desirability or recall bias. Future studies can incorporate objective or behavioral measures where feasible (e.g., teacher reports) to mitigate subjective bias. Last but not least, we did not expect cluster-level differences and did not calculate the sample size based on cluster design. Meanwhile, due to student absences, withdrawals, and dropouts during the study, the final sample failed to meet the originally estimated sample size, potentially reducing statistical power and limiting generalizability.

### 4.5. Suggestions for Improvement of Brief Digital Intervention Design and Implementation

Creating meaningful changes in a single-session intervention is challenging. Drawing on our experiences designing and implementing this program, we identified several directions for improvement. First, we applied patient and public involvement (PPI) principles and co-production with targeted users to enhance the intervention’s relevance and engagement. Unlike in-person counseling or education sessions, users of self-help digital interventions can leave at any time; relevant content is essential for retaining users and sustaining effects (Zhu et al., 2025b). Second, using storytelling may be a good way to engage users. Involving senior students in co-producing the program and telling their stories to help explain the science of mindsets, may make the content more relevant and appealing to younger peers. Third, the timing and setting of delivery are important. Administering classroom surveys for new students can be distracting and may reduce engagement. A better approach may be to provide access to the intervention throughout the research period, so students can use the self-help program when they need it. Fourth, a stronger interaction between parents and children is needed to enhance the impact of the dyadic intervention. Providing opportunities for joint reflection and communication may help both parties better understand each other’s perspectives on mindsets and support needs. Lastly, mixed-methods assessment of the single-session intervention can capture nuanced changes and impacts, providing insights to refine the program for better outcome and improvement. Overall, refining these aspects could help maximize the effectiveness of brief, web-based parent–child interventions in schools.

## 5. Conclusions

In summary, this RCT is the first to evaluate PC-SMILE, an integrated growth mindset intervention for student mental health. This study offers initial evidence of the implementation and potential of a web-based, single-session parent–child mindset intervention among secondary school students in a real-world school setting. Although the current trial did not provide robust evidence that PC-SMILE was superior to the comparison conditions, some improvements were observed, particularly for anxiety and depression symptoms, hopelessness, parent–child relationships, and among adolescents with higher baseline symptoms or girls. High parental acceptance suggests a promise for further development. Future work should focus on boosting child engagement, allowing repeated access to materials, incorporating more interactive parent–child components, and adapting the program for younger and more diverse populations to maximize impact and scalability.

## Figures and Tables

**Table 1:** Baseline characteristics of participants.

Variables/Child	PC-SMILE (n = 126)	C-SMILE (n = 138)	Control (n = 126)	Overall (n = 390)	*p* Value
Age					0.022
Mean (SD)	12.3 (0.5)	12.3 (0.6)	12.5 (0.8)	12.4 (0.6)	
Range	12.0 to 14.0	12.0 to 15.0	12.0 to 15.0	12.0 to 15.0	
Gender, n (%)					0.28
Male	49 (38.9%)	57 (41.3%)	61 (48.4%)	167 (42.8%)	
Female	77 (61.1%)	81 (58.7%)	65 (51.6%)	223 (57.2%)	
Ethnicity, n (%)					0.46
Chinese	123 (97.6%)	134 (97.1%)	125 (99.2%)	382 (97.9%)	
Other	3 (2.4%)	4 (2.9%)	1 (0.8%)	8 (2.1%)	
If an only child, n (%)					0.46
Yes	40 (31.7%)	35 (25.4%)	39 (31.0%)	114 (29.2%)	
No	86 (68.3%)	103 (74.6%)	87 (69.0%)	276 (70.8%)	
If living with parents, n (%)					0.91
Living with both parents	106 (84.1%)	119 (86.2%)	112 (88.9%)	337 (86.4%)	
Living with mother	13 (10.3%)	11 (8.0%)	10 (7.9%)	34 (8.7%)	
Living with father	3 (2.4%)	3 (2.2%)	1 (0.8%)	7 (1.8%)	
Living with neither parent	4 (3.2%)	5 (3.6%)	3 (2.4%)	12 (3.1%)	
Mindset about intelligence (1–6), mean (SD)	3.3 (1.2)	3.6 (1.3)	3.6 (1.2)	3.5 (1.2)	0.056
Mindset about failure (1–6), mean (SD)	3.0 (0.8)	3.0 (0.9)	3.0 (0.9)	3.0 (0.9)	0.85
Mindset about emotions (1–6), mean (SD)	3.1 (1.1)	3.2 (1.3)	3.2 (1.4)	3.2 (1.3)	0.86
RCADS-25: child version-overall (0–75), mean (SD)	18.8 (13.6)	19.2 (13.3)	19.9 (14.9)	19.3 (13.9)	0.81
RCADS-25: child version-anxiety (0–45), mean (SD)	11.8 (8.8)	11.8 (8.2)	12.1 (8.9)	11.9 (8.6)	0.95
RCADS-25: child version-depression (0–30), mean (SD)	7.0 (5.5)	7.4 (5.8)	7.8 (6.7)	7.4 (6.0)	0.57
Hopelessness (1–5), mean (SD)	2.3 (1.0)	2.2 (1.0)	2.1 (1.1)	2.2 (1.0)	0.47
Mental well-being (7–35), mean (SD)	23.9 (5.5)	24.3 (5.5)	23.6 (6.2)	23.9 (5.7)	0.53
Parent–child interactions (0–7), mean (SD)	3.4 (1.9)	3.3 (1.9)	3.6 (2.1)	3.4 (2.0)	0.49
Parent–child relationships (1–4), mean (SD)	2.9 (0.8)	2.9 (0.8)	3.0 (0.8)	2.9 (0.8)	0.54
Academic self-efficacy (1–6), mean (SD)	4.2 (1.0)	4.1 (1.0)	4.2 (1.2)	4.2 (1.1)	0.55
Perceived parent performance (vs. learning) orientation (1–6), mean (SD)	3.3 (0.7)	3.4 (0.7)	3.5 (0.6)	3.4 (0.7)	0.28
Variables/parent	PC-SMILE (n = 126)	C-SMILE (n = 123)	Control (n = 121)	Overall (n = 370)	
Relation with child, n (%)					0.79
Mother	100 (79.4%)	101 (82.1%)	101 (83.5%)	302 (81.6%)	
Father	24 (19.0%)	19 (15.4%)	19 (15.7%)	62 (16.8%)	
Other caregivers	2 (1.6%)	3 (2.4%)	1 (0.8%)	6 (1.6%)	
Ethnicity, n (%)					0.14
Chinese	124 (98.4%)	123 (100.0%)	121 (100.0%)	368 (99.5%)	
Other	2 (1.6%)	0 (0%)	0 (0%)	2 (0.5%)	
Age range, n (%)					0.16
<30	1 (0.8%)	0 (0%)	2 (1.7%)	3 (0.8%)	
30–40	41 (32.5%)	27 (22.0%)	27 (22.3%)	95 (25.7%)	
41–50	74 (58.7%)	79 (64.2%)	73 (60.3%)	226 (61.1%)	
51–60	10 (7.9%)	17 (13.8%)	16 (13.2%)	43 (11.6%)	
61–70	0 (0%)	0 (0%)	2 (1.7%)	2 (0.5%)	
>70	0 (0%)	0 (0%)	1 (0.8%)	1 (0.3%)	
Marital status, n (%)					0.71
Single	5 (4.0%)	2 (1.6%)	1 (0.8%)	8 (2.2%)	
Divorced	7 (5.6%)	9 (7.3%)	7 (5.8%)	23 (6.2%)	
Married/Cohabiting	106 (84.1%)	106 (86.2%)	107 (88.4%)	319 (86.2%)	
Widowed	0 (0%)	1 (0.8%)	1 (0.8%)	2 (0.5%)	
Declined to disclose	8 (6.3%)	5 (4.1%)	5 (4.1%)	18 (4.9%)	
Education level, n (%)					0.49
Primary and below	1 (0.8%)	3 (2.4%)	5 (4.1%)	9 (2.4%)	
Middle School	75 (59.5%)	69 (56.1%)	72 (59.5%)	216 (58.4%)	
College/University and above	50 (39.7%)	51 (41.5%)	44 (36.4%)	145 (39.2%)	
Employment Status, n (%)					0.43
Full-time job	60 (47.6%)	61 (49.6%)	55 (45.5%)	176 (47.6%)	
Part-time job	30 (23.8%)	22 (17.9%)	20 (16.5%)	72 (19.5%)	
Unemployed/At home	36 (28.6%)	40 (32.5%)	46 (38.0%)	122 (33.0%)	
Household Monthly Income (HKD), n (%)					0.92
<3000	1 (0.8%)	3 (2.4%)	2 (1.7%)	6 (1.6%)	
3000–12,000	13 (10.3%)	15 (12.2%)	13 (10.7%)	41 (11.1%)	
12,001–20,000	27 (21.4%)	23 (18.7%)	28 (23.1%)	78 (21.1%)	
20,001–35,000	34 (27.0%)	30 (24.4%)	37 (30.6%)	101 (27.3%)	
35,001–50,000	21 (16.7%)	23 (18.7%)	20 (16.5%)	64 (17.3%)	
50,001–90,000	22 (17.5%)	18 (14.6%)	12 (9.9%)	52 (14.1%)	
>90,000	8 (6.3%)	11 (8.9%)	9 (7.4%)	28 (7.6%)	
Mindset about intelligence (1–6), mean (SD)	3.2 (1.0)	3.3 (1.2)	3.3 (1.2)	3.2 (1.1)	0.69
Mindset about failure (1–6), mean (SD)	2.7 (0.6)	2.6 (0.7)	2.7 (0.8)	2.6 (0.7)	0.57
Mindset about emotions (1–6), mean (SD)	2.4 (0.7)	2.4 (0.7)	2.4 (0.7)	2.4 (0.7)	0.66
RCADS-25: parent version-overall (0–75), mean (SD)	11.1 (7.5)	12.0 (7.1)	11.7 (7.5)	11.6 (7.4)	0.68
RCADS-25: parent version-anxiety (0–45), mean (SD)	5.7 (4.4)	6.0 (4.2)	6.1 (4.3)	5.9 (4.3)	0.76
RCADS-25: parent version-depression (0–30), mean (SD)	5.4 (3.7)	5.9 (3.7)	5.6 (4.1)	5.6 (3.8)	0.57
Mental well-being (7–35), mean (SD)	25.2 (4.8)	25.4 (3.6)	25.8 (4.8)	25.5 (4.4)	0.55
Parent–child relationships (1–4), mean (SD)	3.1 (0.5)	3.0 (0.5)	3.2 (0.6)	3.1 (0.6)	0.27

**Table 2:** Generalized estimating equation results for child participants, intention-to-treat population (estimated marginal means (standard error)).

Outcome Variables	Baseline	2-Week Follow-Up	3-Month Follow-Up	*p*-Value (Baseline vs. 2-Week Follow-Up)	*p*-Value (Baseline vs. 3-Month Follow-Up)	*p*-Value (2-Week vs. 3-Month Follow-Up)
RCADS-25: child version-overall						
PC-SMILE	18.2 (1.5)	14.9 (1.4)	16.9 (1.6)	<0.001	0.23	0.12
C-SMILE	16.8 (1.5)	15.7 (1.6)	16.4 (1.6)	0.38	0.72	0.49
Control	19.8 (1.7)	17.5 (1.6)	18.3 (1.6)	0.06	0.15	0.52
*P*_PC-SMILE vs. C-SMILE_	0.42	0.66	0.81	*p*-value (interaction)	0.72	
*P*_PC-SMILE vs. Control_	0.38	0.11	0.46			
*P*_C-SMILE vs. Control_	0.11	0.33	0.32			
RCADS-25: child version-anxiety						
PC-SMILE	11.4 (1.0)	8.9 (0.9)	10.4 (1.0)	<0.001	0.11	0.08
C-SMILE	10.4 (1.0)	9.5 (1.0)	10.1 (1.0)	0.22	0.63	0.35
Control	11.9 (1.0)	10.6 (1.0)	10.9 (1.0)	0.07	0.13	0.68
*P*_PC-SMILE vs. C-SMILE_	0.36	0.58	0.83	*p*-value (interaction)	0.53	
*P*_PC-SMILE vs. Control_	0.66	0.08	0.63			
*P*_C-SMILE vs. Control_	0.19	0.33	0.48			
RCADS-25: child version-depression						
PC-SMILE	7.5 (0.2)	6.7 (0.2)	7.2 (0.3)	0.014	0.54	0.21
C-SMILE	4.9 (0.3)	4.7 (0.3)	4.9 (0.3)	0.72	0.99	0.49
Control	9.8 (0.3)	8.9 (0.3)	9.6 (0.2)	0.14	0.62	0.050
*P*_PC-SMILE vs. C-SMILE_	<0.001	<0.001	<0.001	*p*-value (interaction)	<0.001	
*P*_PC-SMILE vs. Control_	<0.001	<0.001	<0.001			
*P*_C-SMILE vs. Control_	<0.001	<0.001	<0.001			
Hopelessness						
PC-SMILE	2.3 (0.1)	2.1 (0.1)	2.2 (0.1)	0.031	0.31	0.18
C-SMILE	2.1 (0.1)	2.1 (0.1)	2.1 (0.1)	0.94	0.95	0.89
Control	2.2 (0.1)	2.2 (0.1)	2.2 (0.1)	0.43	0.83	0.56
*P*_PC-SMILE vs. C-SMILE_	0.11	0.91	0.40	*p*-value (interaction)	0.31	
*P*_PC-SMILE vs. Control_	0.31	0.29	0.83			
*P*_C-SMILE vs. Control_	0.59	0.28	0.53			
Mental well-being						
PC-SMILE	24.1 (0.3)	25.0 (0.4)	24.6 (0.2)	0.14	0.13	0.47
C-SMILE	25.4 (0.3)	25.3 (0.1)	24.8 (0.4)	0.74	0.35	0.29
Control	21.2 (0.4)	21.8 (0.4)	21.2 (0.3)	0.37	0.92	0.26
*P*_PC-SMILE vs. C-SMILE_	<0.001	0.40	0.41	*p*-value (interaction)	0.06	
*P*_PC-SMILE vs. Control_	<0.001	<0.001	<0.001			
*P*_C-SMILE vs. Control_	<0.001	<0.001	<0.001			
Parent–child interactions						
PC-SMILE	3.38 (0.07)	3.43 (0.10)	3.54 (0.10)	0.71	0.25	0.55
C-SMILE	3.33 (0.07)	3.37 (0.05)	3.17 (0.06)	0.78	0.17	0.012
Control	2.90 (0.13)	2.87 (0.08)	2.82 (0.10)	0.87	0.70	0.50
*P*_PC-SMILE vs. C-SMILE_	0.74	0.59	0.008	*p*-value (interaction)	0.50	
*P*_PC-SMILE vs. Control_	0.009	<0.001	<0.001			
*P*_C-SMILE vs. Control_	<0.001	<0.001	<0.001			
Parent–child relationships						
PC-SMILE	2.8 (0.08)	2.9 (0.08)	2.9 (0.08)	0.005	0.002	0.83
C-SMILE	2.8 (0.09)	2.8 0.09)	2.8 (0.09)	0.52	0.92	0.58
Control	2.8 (0.09)	2.9 (0.09)	2.8 (0.09)	0.22	0.70	0.14
*P*_PC-SMILE vs. C-SMILE_	0.92	0.29	0.20	*p*-value (interaction)	0.20	
*P*_PC-SMILE vs. Control_	0.56	0.83	0.28			
*P*_C-SMILE vs. Control_	0.65	0.43	0.88			
Academic self-efficacy						
PC-SMILE	3.9 (0.06)	3.9 (0.06)	3.9 (0.03)	0.95	0.95	0.97
C-SMILE	4.3 (0.06)	4.4 (0.05)	4.3 (0.06)	0.68	0.58	0.21
Control	3.9 (0.07)	3.8 (0.08)	3.9 (0.05)	0.74	0.79	0.51
*P*_PC-SMILE vs. C-SMILE_	<0.001	<0.001	<0.001	*p*-value (interaction)	0.49	
*P*_PC-SMILE vs. Control_	0.06	0.17	00.44			
*P*_C-SMILE vs. Control_	<0.001	<0.001	<0.001			
Perceived parent performance (vs. learning) orientation						
PC-SMILE	3.4 (0.07)	3.4 (0.07)	3.3 (0.07)	0.45	0.33	0.87
C-SMILE	3.5 (0.07)	3.3 (0.07)	3.4 (0.07)	0.012	0.06	0.50
Control	3.5 (0.07)	3.4 (0.07)	3.4 (0.07)	0.11	0.006	0.35
*P*_PC-SMILE vs. C-SMILE_	0.32	0.69	0.84	*p*-value (interaction)	0.58	
*P*_PC-SMILE vs. Control_	0.12	0.31	0.66			
*P*_C-SMILE vs. Control_	0.56	0.15	0.81			

**Table 3:** Effect sizes (Cohen’s d (standard error)) of the treatments on child participants, intention-to-treat population.

Outcome Variables	2-Week Follow-Up, Cohen’s *d* (SE)	3-Month Follow-Up, Cohen’s *d* (SE)
RCADS-25: child version-overall		
PC-SMILE vs. C-SMILE	−0.16 (0.15)	−0.07 (0.15)
PC-SMILE vs. Control	−0.07 (0.14)	0.01 (0.15)
C-SMILE vs. Control	0.08 (0.18)	0.08 (0.18)
RCADS-25: child version-anxiety		
PC-SMILE vs. C-SMILE	−0.19 (0.15)	−0.09 (0.16)
PC-SMILE vs. Control	−0.15 (0.15)	−0.01 (0.15)
C-SMILE vs. Control	0.04 (0.18)	0.08 (0.19)
RCADS-25: child version-depression		
PC-SMILE vs. C-SMILE	−0.10 (0.09)	−0.05 (0.11)
PC-SMILE vs. Control	0.02 (0.08)	−0.01 (0.10)
C-SMILE vs. Control	0.12 (0.09)	0.04 (0.10)
Hopelessness		
PC-SMILE vs. C-SMILE	−0.19 (0.16)	−0.09 (0.15)
PC-SMILE vs. Control	−0.26 (0.15)	−0.10 (0.15)
C-SMILE vs. Control	−0.07 (0.18)	−0.01 (0.18)
Mental well-being		
PC-SMILE vs. C-SMILE	0.17 (0.12)	0.18 (0.10)
PC-SMILE vs. Control	0.05 (0.14)	0.07 (0.06)
C-SMILE vs. Control	−0.12 (0.10)	−0.11 (0.09)
Parent–child interactions		
PC-SMILE vs. C-SMILE	0.01 (0.08)	0.16 (0.08)
PC-SMILE vs. Control	0.04 (0.09)	0.13 (0.08)
C-SMILE vs. Control	0.03 (0.05)	−0.04 (0.05)
Parent–child relationships		
PC-SMILE vs. C-SMILE	0.14 (0.14)	0.17 (0.14)
PC-SMILE vs. Control	0.10 (0.14)	0.21 (0.11)
C-SMILE vs. Control	−0.04 (0.18)	0.04 (0.16)
Academic self-efficacy		
PC-SMILE vs. C-SMILE	−0.04 (0.13)	0.06 (0.11)
PC-SMILE vs. Control	0.04 (0.15)	−0.03 (0.11)
C-SMILE vs. Control	0.08 (0.13)	−0.08 (0.12)
Perceived parent performance (vs. learning) orientation		
PC-SMILE vs. C-SMILE	0.18 (0.16)	0.10 (0.16)
PC-SMILE vs. Control	0.07 (0.16)	0.14 (0.16)
C-SMILE vs. Control	−0.10 (0.18)	0.04 (0.19)

Note. Effect size values were calculated by subtracting the latter group’s mean gain score from the former group’s mean gain score for each outcome from baseline to the 2-week and 3-month follow-ups, then dividing by the pooled standard deviations of all participants at baseline.

**Table 4:** Feedback on the intervention.

Variables/Child	PC-SMILE (n = 125), n (%)	C-SMILE (n = 138), n (%)	Overall (n = 263), n (%)
Like this course	66 (52.8%)	74 (53.6%)	140 (53.2%)
Understand this course	86 (68.8%)	84 (60.9%)	170 (64.6%)
This course is useful	86 (68.8%)	87 (63.0%)	173 (65.8%)
Attitude towards adversity improved after this course	72 (57.6%)	83 (60.1%)	155 (58.9%)
Complete this course very seriously	87 (69.6%)	98 (71.0%)	185 (70.3%)
Recommend this course to others	73 (58.4%)	86 (62.3%)	159 (60.5%)
This course is interesting	72 (57.6%)	71 (51.4%)	143 (54.4%)
Agree with this course	86 (68.8%)	87 (63.0%)	173 (65.8%)
More confident towards challenges in life after this course	73 (58.4%)	79 (57.2%)	152 (57.8%)
Burden in joining this course	7 (5.6%)	13 (9.4%)	20 (7.6%)
Affect other arrangement due to joining this course	8 (6.4%)	15 (10.9%)	23 (8.7%)
General acceptance of this course	85 (68.0%)	98 (71.0%)	183 (69.6%)
Variables/parent	PC-SMILE (n = 124), n (%)		
Like this course	95 (76.6%)		
Understand this course	109 (87.9%)		
This course is useful	109 (87.9%)		
This course provides some references to help me communicate with my child in daily life	113 (91.1%)		
Complete this course very seriously	117 (94.4%)		
Recommend this course to other parents	100 (80.6%)		
This course is interesting	87 (70.2%)		
Agree with this course	112 (90.3%)		
More confident in helping my child face the challenges in life after this course	92 (74.2%)		
Burden in joining this course	7 (5.6%)		
Affect other arrangement due to joining this course	10 (8.1%)		
General acceptance of this course	105 (84.7%)		

## Data Availability

Data are not publicly available; only anonymized data may be made available upon reasonable request for appropriate research purposes.

## Supplementary Materials

The following supporting information can be downloaded at: https://www.mdpi.com/article/10.3390/ejihpe16060084/s1, Table S1: Generalized estimating equation results for child participants, per-protocol population (estimated marginal means (standard error)); Table S2: Generalized estimating equation results for parent participants, intention-to-treat population (estimated marginal means (standard error)); Table S3: Effect sizes (Cohen’s *d* (standard error)) of the treatments on parent participants, intention-to-treat population; Table S4: Moderator effects of baseline children’s depression and anxiety symptoms on the treatment effects; Table S5: Moderation effects of gender on the treatment effects; Figure S1: Child gender distribution across the PC-SMILE, C-SMILE, and control groups at baseline; Figure S2: Only-child status across the PC-SMILE, C-SMILE, and control groups at baseline; Figure S3: Parent education level across the PC-SMILE, C-SMILE, and control groups at baseline; Figure S4. Household monthly income across the PC-SMILE, C-SMILE, and control groups at baseline.

## Abbreviations

The following abbreviations are used in this manuscript:

CONSORT	Consolidated Standards of Reporting Trials
C-SMILE	Child single-session intervention on mindsets of intelligence, failure, and emotion
DMHI	Digital mental health interventions
GEE	Generalized estimating equations
MDASS	Mindset of Depression, Anxiety and Stress Scale
PC-SMILE	Parent and child single-session intervention on mindsets of intelligence, failure, and emotion
PPI	Patient and public involvement
QICC	Corrected Quasi Likelihood under Independence Model Criterion
RCT	Randomized controlled trial
RCADS-25	25-item Revised Children’s Anxiety and Depression Scale
We-SMILE	Web-based single-session intervention of mindsets on intelligence, failure, and emotion
