# Supplementary material for: Effects of a Digital Parent–Child Single-Session Growth Mindset Intervention on Adolescent Depression and Anxiety Symptoms: A Three-Arm Waitlist Randomized Controlled Trial
Source: Eur J Investig Health Psychol Educ. 2026 Jun 17;16(6):84. doi: 10.3390/ejihpe16060084 (PMC13297808; doi:10.3390/ejihpe16060084)
Supplement: Supplementary file 1 [file ejihpe-16-00084-s001.zip › Supplementary Figure S1-4.pdf]

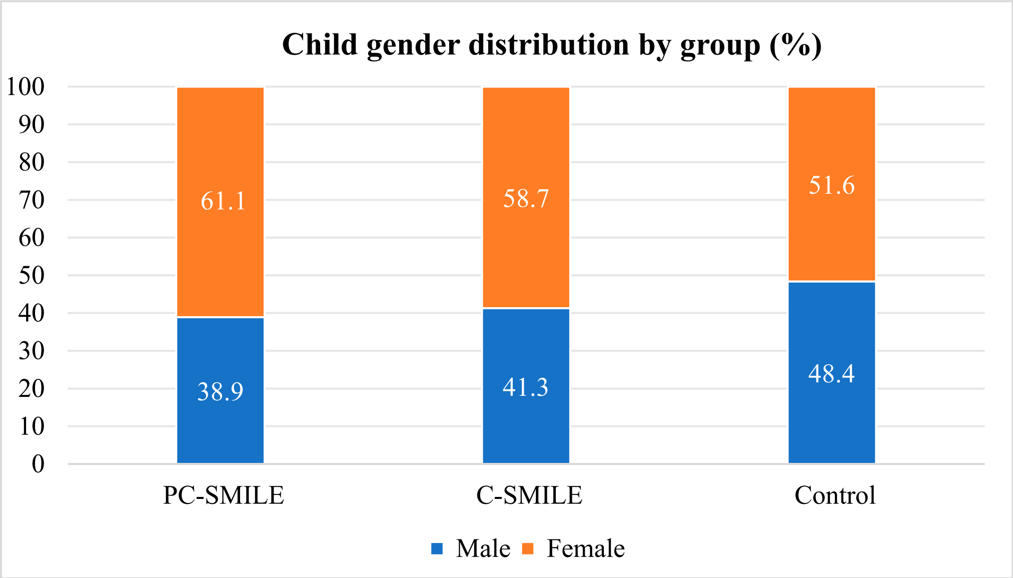

Figure S1. Child gender distribution across the PC-SMILE, C-SMILE, and control groups at baseline. Bars show the percentage of male and female participants in each group.

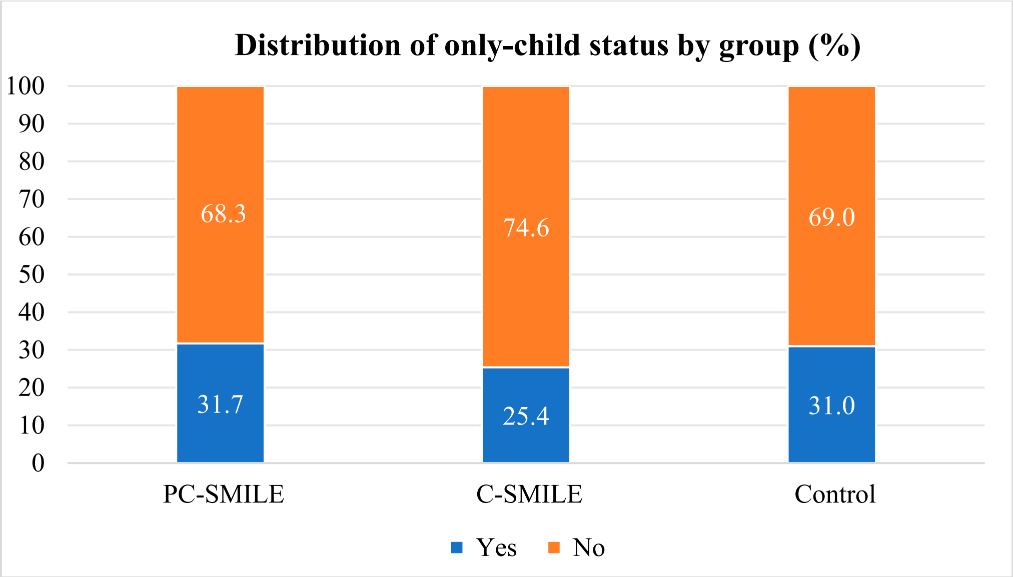

Figure S2. Only-child status across the PC-SMILE, C-SMILE, and control groups at baseline. Bars show the percentage of participants who were only children and those who were not in each group.

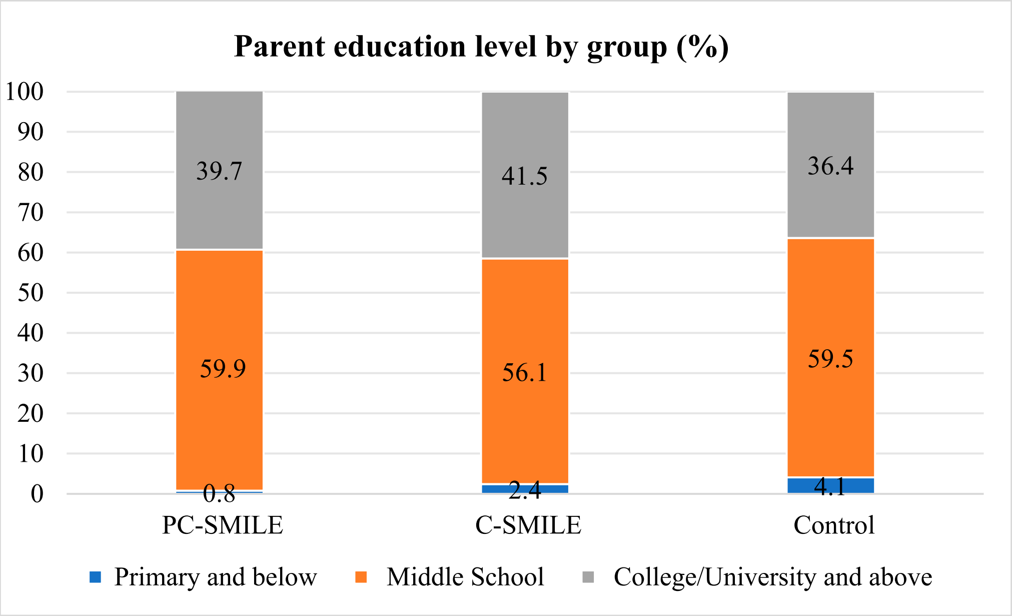

Figure S3. Parent education level across the PC-SMILE, C-SMILE, and control groups at baseline. Bars show the percentage of parents with primary education or below, middle school education, and college/university education or above in each group.

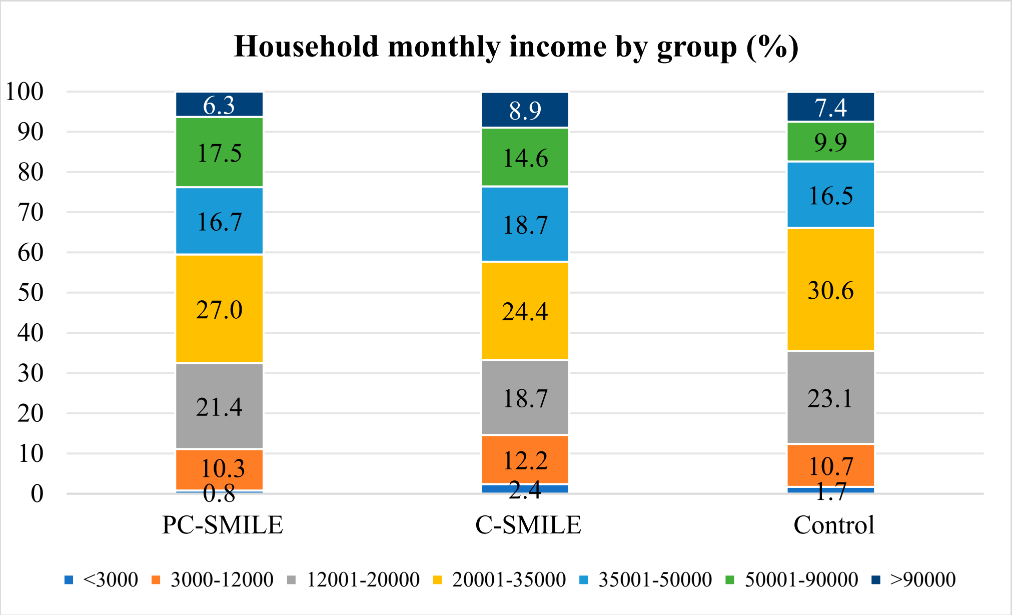

Figure S4. Household monthly income across the PC-SMILE, C-SMILE, and control groups at baseline. Bars show the percentage distribution of household monthly income categories in each group.
