# Supplementary material for: Effects of a Digital Parent–Child Single-Session Growth Mindset Intervention on Adolescent Depression and Anxiety Symptoms: A Three-Arm Waitlist Randomized Controlled Trial
Source: Eur J Investig Health Psychol Educ. 2026 Jun 17;16(6):84. doi: 10.3390/ejihpe16060084 (PMC13297808; doi:10.3390/ejihpe16060084)
Supplement: Supplementary file 1 [file ejihpe-16-00084-s001.zip › Supplementary Table S1-5_revised.pdf]

Table S1. Generalized estimating equation results for child participants, per-protocol population (estimated marginal means (standard error)).

Outcome variables	Baseline	2-week follow-up	3-month follow-up	*P*-value (baseline vs 2-week follow-up)	*P*-value (baseline vs 3-month follow-up)	*P*-value (2-week vs 3-month follow-up)
**RCADS-25: child version-overall**						
PC-SMILE	17.4 (2.7)	15.5 (2.5)	15.7 (2.5)	.19	.25	.87
C-SMILE	20.7 (2.4)	19.2 (2.6)	20.1 (2.6)	.21	.60	.47
$P_{\text{PC-SMILE vs C-SMILE}}$	.34	.28	.21	*P*-value (interaction)	.85	
**RCADS-25: child version-anxiety**						
PC-SMILE	11.2 (1.7)	9.5 (1.6)	9.7 (1.6)	.08	.08	.89
C-SMILE	12.5 (1.5)	11.8 (1.7)	12.3 (1.6)	.33	.76	.51
$P_{\text{PC-SMILE vs C-SMILE}}$	.55	.33	.25	*P*-value (interaction)	.55	
**RCADS-25: child version-depression**						
PC-SMILE	6.2 (1.1)	5.9 (1.0)	6.1 (1.0)	.65	.82	.86
C-SMILE	8.2 (1.0)	7.5 (1.0)	7.8 (1.0)	.17	.46	.49
$P_{\text{PC-SMILE vs C-SMILE}}$	.16	.26	.21	*P*-value (interaction)	.86	
**Hopelessness**						
PC-SMILE	2.2 (0.2)	2.0 (0.2)	2.1 (0.2)	.26	.44	.53
C-SMILE	2.1 (0.2)	2.2 (0.2)	2.1 (0.2)	.47	.47	.95
$P_{\text{PC-SMILE vs C-SMILE}}$	.75	.53	.82	*P*-value (interaction)	.37	
**Mental well-being**						
PC-SMILE	25.0 (0.3)	25.0 (0.4)	25.1 (0.5)	.98	.84	.86
C-SMILE	23.6 (0.1)	23.9 (0.1)	24.2 (0.2)	.59	.30	.55
$P_{\text{PC-SMILE vs C-SMILE}}$	.19	.38	.43	*P*-value (interaction)	.90	
**Parent–child interactions**						
PC-SMILE	3.6 (0.3)	3.4 (0.3)	3.5 (0.3)	.39	.49	.77
C-SMILE	3.5 (0.3)	3.6 (0.3)	3.3 (0.3)	.47	.40	.013
$P_{\text{PC-SMILE vs C-SMILE}}$	.79	.59	.69	*P*-value (interaction)	.34	
**Parent–child relationships**						
PC-SMILE	3.1 (0.03)	3.2 (0.02)	3.1 (0.04)	.25	1.0	.27

C-SMILE	2.8 (0.06)	2.9 (0.03)	3.0 (0.04)	.21	.06	.33
$P_{\text{PC-SMILE vs C-SMILE}}$	.14	.13	.57	*P*-value (interaction)	.17	
**Academic self-efficacy**						
PC-SMILE	4.5 (0.2)	4.2 (0.2)	4.3 (0.2)	.10	.18	.49
C-SMILE	3.9 (0.2)	4.1 (0.1)	4.1 (0.2)	.17	.04	.40
$P_{\text{PC-SMILE vs C-SMILE}}$	.006	.36	.40	*P*-value (interaction)	.042	
**Perceived parent performance (vs. learning) orientation**						
PC-SMILE	3.3 (0.1)	3.3 (0.1)	3.2 (0.1)	.65	.49	.74
C-SMILE	3.3 (0.1)	3.2 (0.1)	3.2 (0.1)	.48	.85	.56
$P_{\text{PC-SMILE vs C-SMILE}}$	.76	.62	.10	*P*-value (interaction)	.81	

Table S2. Generalized estimating equation results for parent participants, intention-to-treat population (estimated marginal means (standard error))

Outcome variables	Baseline	3-month follow-up	*P*-value (baseline vs 3-month follow-up)	
**RCADS-25: parent version-overall**				
PC-SMILE	11.6 (0.6)	35.5 (0.7)	< .001	
C-SMILE	11.7 (0.8)	36.8 (0.9)	< .001	
Control	12.4 (0.8)	37.8 (0.9)	< .001	
$P_{\text{PC-SMILE vs C-SMILE}}$	.91	.26	*P*-value (interaction)	.21
$P_{\text{PC-SMILE vs Control}}$	.43	.031		
$P_{\text{C-SMILE vs Control}}$	.51	.40		
**RCADS-25: parent version-anxiety**				
PC-SMILE	6.0 (0.4)	20.6 (0.4)	< .001	
C-SMILE	5.9 (0.5)	21.0 (0.5)	< .001	
Control	6.5 (0.4)	21.9 (0.5)	< .001	
$P_{\text{PC-SMILE vs C-SMILE}}$	.87	.51	*P*-value (interaction)	.29
$P_{\text{PC-SMILE vs Control}}$	.39	.038		
$P_{\text{C-SMILE vs Control}}$	.33	.22		
**RCADS-25: parent version-depression**				
PC-SMILE	5.6 (0.3)	15.0 (0.3)	< .001	

C-SMILE	5.8 (0.4)	15.8 (0.5)	< .001	
Control	5.9 (0.4)	16.0 (0.4)	< .001	
$P_{\text{PC-SMILE vs C-SMILE}}$	.68	.15	*P*-value (interaction)	.22
$P_{\text{PC-SMILE vs Control}}$	.57	.06		
$P_{\text{C-SMILE vs Control}}$	.87	.82		
**Mental well-being**				
PC-SMILE	24.5 (0.4)	25.3 (0.5)	.017	
C-SMILE	24.6 (0.5)	25.0 (0.6)	.36	
Control	24.9 (0.5)	24.9 (0.5)	.87	
$P_{\text{PC-SMILE vs C-SMILE}}$	.93	.65	*P*-value (interaction)	.26
$P_{\text{PC-SMILE vs Control}}$	.54	.50		
$P_{\text{C-SMILE vs Control}}$	.60	.87		
**Parent–child relationships**				
PC-SMILE	2.9 (0.05)	2.9 (0.05)	.10	
C-SMILE	2.9 (0.07)	2.9 (0.07)	.64	
Control	3.0 (0.06)	3.0 (0.06)	.83	
$P_{\text{PC-SMILE vs C-SMILE}}$	.58	.63	*P*-value (interaction)	.38
$P_{\text{PC-SMILE vs Control}}$	.51	.21		
$P_{\text{C-SMILE vs Control}}$	.27	.52		

Table S3. Effect sizes (Cohen's *d* (standard error)) of the treatments on parent participants, intention-to-treat population.

Outcome variables	3-month follow-up, Cohen's *d* (SE)
**RCADS-25: parent version-overall**	
PC-SMILE vs C-SMILE	-0.16 (0.18)
PC-SMILE vs Control	-0.21 (0.17)
C-SMILE vs Control	-0.05 (0.21)
**RCADS-25: parent version-anxiety**	
PC-SMILE vs C-SMILE	-0.13 (0.17)
PC-SMILE vs Control	-0.20 (0.17)
C-SMILE vs Control	-0.07 (0.21)
**RCADS-25: parent version-depression**	
PC-SMILE vs C-SMILE	-0.17 (0.18)
PC-SMILE vs Control	-0.19 (0.16)
C-SMILE vs Control	-0.02 (0.21)
**Mental well-being**	
PC-SMILE vs C-SMILE	0.09 (0.18)
PC-SMILE vs Control	0.19 (0.16)

C-SMILE vs Control	0.10 (0.22)
**Parent–child relationships**	
PC-SMILE vs C-SMILE	-0.15 (0.16)
PC-SMILE vs Control	-0.09 (0.15)
C-SMILE vs Control	0.06 (0.20)

[1] Effect size values were calculated by subtracting the latter group's mean gain score from the former group's mean gain score for each outcome from baseline to the 3-month follow-up, then dividing by the pooled standard deviations of all participants at baseline.

Table S4. Moderator effects of baseline children's depression and anxiety symptoms on the treatment effects.

Outcome variables	Baseline	2-week follow-up	3-month follow-up	*P*-value (baseline vs 2-week follow-up)	*P*-value (baseline vs 3-month follow-up)	*P*-value (2-week vs 3-month follow-up)
**RCADS-25: child version-overall**						
High RCADS-25 level						
PC-SMILE	32.5 (1.7)	24.3 (1.8)	29.9 (2.3)	<.001	.17	.012
C-SMILE	30.6 (1.7)	23.9 (1.9)	26.3 (1.9)	<.001	<.001	.08
Control	35.0 (1.9)	28.6 (2.2)	31.0 (2.1)	.003	.039	.27
$P_{\text{PC-SMILE vs C-SMILE}}$	.30	.86	.17	*P*-value (interaction)	.80	
$P_{\text{PC-SMILE vs Control}}$	.23	.09	.72			
$P_{\text{C-SMILE vs Control}}$	.036	.08	.06			
Low RCADS-25 level						
PC-SMILE	7.0 (0.6)	8.1 (0.9)	8.1 (1.0)	.20	.26	.95
C-SMILE	8.0 (0.8)	11.4 (1.6)	11.8 (1.4)	.07	.011	.76
Control	7.4 (0.8)	9.1 (1.1)	8.9 (1.1)	.08	.039	.87
$P_{\text{PC-SMILE vs C-SMILE}}$	.28	.09	.034	*P*-value (interaction)	.60	
$P_{\text{PC-SMILE vs Control}}$	.67	.52	.55			
$P_{\text{C-SMILE vs Control}}$	.55	.26	.10			
**RCADS-25: child version-anxiety**						
High RCADS-25 level						
PC-SMILE	20.7 (1.1)	14.8 (1.2)	18.7 (1.5)	<.001	.10	.010
C-SMILE	19.4 (1.1)	15.1 (1.2)	16.4 (1.2)	<.001	<.001	.10
Control	21.4 (1.1)	17.7 (1.4)	18.8 (1.3)	.006	.043	.39
$P_{\text{PC-SMILE vs C-SMILE}}$	.30	.87	.19	*P*-value (interaction)	.60	
$P_{\text{PC-SMILE vs Control}}$	.56	.06	.92			
$P_{\text{C-SMILE vs Control}}$	.11	.11	.12			
Low RCADS-25 level						

PC-SMILE	4.6 (0.4)	4.9 (0.5)	5.0 (0.6)	.48	.40	.89
C-SMILE	5.0 (0.5)	6.7 (1.0)	7.4 (0.9)	.12	.015	.44
Control	4.6 (0.4)	5.6 (0.7)	5.4 (0.6)	.10	.11	.79
$P_{\text{PC-SMILE vs C-SMILE}}$	.50	.13	.035	*P*-value (interaction)	.48	
$P_{\text{PC-SMILE vs Control}}$	.92	.47	.69			
$P_{\text{C-SMILE vs Control}}$	.57	.34	.06			
**RCADS-25: child version-depression**						
High RCADS-25 level						
PC-SMILE	11.9 (0.7)	9.5 (0.8)	11.3 (0.8)	<.001	.46	.035
C-SMILE	11.2 (0.7)	8.8 (0.8)	9.9 (0.8)	<.001	.019	.09
Control	13.6 (0.9)	10.9 (0.9)	12.1 (0.9)	.003	.08	.20
$P_{\text{PC-SMILE vs C-SMILE}}$	.46	.54	.23	*P*-value (interaction)	.94	
$P_{\text{PC-SMILE vs Control}}$	.08	.22	.49			
$P_{\text{C-SMILE vs Control}}$	.021	.07	.053			
Low RCADS-25 level						
PC-SMILE	2.4 (0.3)	3.2 (0.5)	3.0 (0.5)	.07	.19	.72
C-SMILE	3.0 (0.4)	4.7 (0.8)	4.4 (0.6)	.043	.020	.69
Control	2.8 (0.4)	3.5 (0.6)	3.5 (0.6)	.12	.047	.99
$P_{\text{PC-SMILE vs C-SMILE}}$	.22	.09	.06	*P*-value (interaction)	.83	
$P_{\text{PC-SMILE vs Control}}$	.50	.65	.47			
$P_{\text{C-SMILE vs Control}}$	.64	.22	.26			
**Hopelessness**						
High RCADS-25 level						
PC-SMILE	3.0 (0.1)	2.6 (0.1)	3.0 (0.1)	.005	.77	.008
C-SMILE	2.8 (0.1)	2.5 (0.1)	2.6 (0.1)	.044	.25	.25
Control	3.1 (0.1)	3.0 (0.1)	2.9 (0.1)	.67	.50	.74
$P_{\text{PC-SMILE vs C-SMILE}}$	.13	.59	.09	*P*-value (interaction)	.25	
$P_{\text{PC-SMILE vs Control}}$	.87	.044	.86			
$P_{\text{C-SMILE vs Control}}$	.08	.011	.11			
Low RCADS-25 level						
PC-SMILE	1.7 (0.05)	1.6 (0.07)	1.6 (0.03)	.82	.31	.80
C-SMILE	1.7 (0.07)	1.9 (0.08)	1.9 (0.04)	.17	.14	.49
Control	1.6 (0.07)	1.8 (0.09)	1.8 (0.05)	.11	.023	.61
$P_{\text{PC-SMILE vs C-SMILE}}$	.36	<.001	<.001	*P*-value (interaction)	.004	
$P_{\text{PC-SMILE vs Control}}$	.44	.09	.002			
$P_{\text{C-SMILE vs Control}}$	.09	.26	.11			
**Mental well-being**						
High RCADS-25 level						

PC-SMILE	21.0 (0.7)	22.2 (0.9)	20.9 (0.8)	.08	.96	.12
C-SMILE	22.5 (0.7)	23.2 (0.8)	22.9 (0.8)	.32	.55	.75
Control	20.1 (0.8)	20.7 (0.8)	20.3 (0.9)	.47	.85	.65
$P_{\text{PC-SMILE vs C-SMILE}}$	.09	.42	.07	*P*-value (interaction)	.90	
$P_{\text{PC-SMILE vs Control}}$	.39	.22	.58			
$P_{\text{C-SMILE vs Control}}$	.011	.026	.022			
Low RCADS-25 level						
PC-SMILE	26.8 (0.3)	27.4 (0.6)	27.3 (0.3)	.43	.054	.87
C-SMILE	25.5 (0.2)	24.9 (0.3)	24.1 (0.4)	.018	.005	.19
Control	23.4 (0.5)	23.9 (0.5)	23.3 (0.5)	.59	.92	.57
$P_{\text{PC-SMILE vs C-SMILE}}$	<.001	<.001	<.001	*P*-value (interaction)	<.001	
$P_{\text{PC-SMILE vs Control}}$	<.001	<.001	<.001			
$P_{\text{C-SMILE vs Control}}$	<.001	.048	.31			
**Parent–child interactions**						
High RCADS-25 level						
PC-SMILE	2.8 (0.3)	2.8 (0.2)	2.9 (0.3)	.77	.51	.67
C-SMILE	2.5 (0.2)	2.7 (0.2)	2.5 (0.2)	.37	.87	.40
Control	2.5 (0.3)	2.6 (0.3)	2.7 (0.3)	.71	.56	.80
$P_{\text{PC-SMILE vs C-SMILE}}$	.46	.67	.28	*P*-value (interaction)	.93	
$P_{\text{PC-SMILE vs Control}}$	.53	.55	.40			
$P_{\text{C-SMILE vs Control}}$	.97	.85	.70			
Low RCADS-25 level						
PC-SMILE	2.9 (0.2)	3.0 (0.2)	3.0 (0.2)	.91	.62	.77
C-SMILE	3.5 (0.2)	3.3 (0.3)	3.2 (0.3)	.44	.052	.46
Control	3.6 (0.3)	3.4 (0.3)	3.3 (0.3)	.40	.13	.65
$P_{\text{PC-SMILE vs C-SMILE}}$	.06	.29	.65	*P*-value (interaction)	.41	
$P_{\text{PC-SMILE vs Control}}$	.034	.19	.39			
$P_{\text{C-SMILE vs Control}}$	.83	.87	.74			
**Parent–child relationships**						
High RCADS-25 level						
PC-SMILE	2.6 (0.1)	2.8 (0.1)	2.8 (0.1)	.033	.010	.94
C-SMILE	2.7 (0.1)	2.8 (0.1)	2.7 (0.1)	.40	.63	.66
Control	2.7 (0.1)	2.8 (0.1)	2.7 (0.1)	.52	.87	.53
$P_{\text{PC-SMILE vs C-SMILE}}$	.49	.86	.71	*P*-value (interaction)	.47	
$P_{\text{PC-SMILE vs Control}}$	.44	.88	.54			
$P_{\text{C-SMILE vs Control}}$	.90	.98	.79			
Low RCADS-25 level						

PC-SMILE	3.0 (0.1)	3.1 (0.1)	3.1 (0.1)	.0498	.16	.51
C-SMILE	2.9 (0.1)	2.9 (0.1)	2.9 (0.1)	.68	.73	.47
Control	3.0 (0.1)	3.1 (0.1)	3.0 (0.1)	.40	.42	.09
$P_{\text{PC-SMILE vs C-SMILE}}$	.39	.13	.09	*P*-value (interaction)	.59	
$P_{\text{PC-SMILE vs Control}}$	.91	.78	.37			
$P_{\text{C-SMILE vs Control}}$	.39	.25	.54			
**Academic self-efficacy**						
High RCADS-25 level						
PC-SMILE	4.1 (0.2)	3.9 (0.2)	3.9 (0.2)	.21	.30	.99
C-SMILE	4.0 (0.2)	4.1 (0.2)	4.1 (0.2)	.15	.53	.49
Control	3.9 (0.2)	3.9 (0.2)	4.0 (0.2)	.85	.28	.46
$P_{\text{PC-SMILE vs C-SMILE}}$	.47	.30	.62	*P*-value (interaction)	.28	
$P_{\text{PC-SMILE vs Control}}$	.21	.80	.69			
$P_{\text{C-SMILE vs Control}}$	.61	.23	.93			
Low RCADS-25 level						
PC-SMILE	4.2 (0.1)	4.3 (0.1)	4.3 (0.1)	.41	.36	.92
C-SMILE	4.1 (0.1)	4.0 (0.2)	3.9 (0.2)	.55	.10	.40
Control	4.4 (0.2)	4.2 (0.2)	4.2 (0.2)	.34	.24	.97
$P_{\text{PC-SMILE vs C-SMILE}}$	.71	.18	.06	*P*-value (interaction)	.38	
$P_{\text{PC-SMILE vs Control}}$	.27	.89	.95			
$P_{\text{C-SMILE vs Control}}$	.17	.31	.11			
**Perceived parent performance (vs. learning) orientation**						
High RCADS-25 level						
PC-SMILE	3.3 (0.1)	3.3 (0.1)	3.2 (0.1)	.30	.20	.72
C-SMILE	3.4 (0.1)	3.3 (0.1)	3.3 (0.1)	.05	.11	.60
Control	3.6 (0.1)	3.5 (0.1)	3.5 (0.1)	.12	.06	.97
$P_{\text{PC-SMILE vs C-SMILE}}$	.43	.89	.45	*P*-value (interaction)	.96	
$P_{\text{PC-SMILE vs Control}}$	.06	.08	.08			
$P_{\text{C-SMILE vs Control}}$	.14	.09	.22			
Low RCADS-25 level						
PC-SMILE	3.2 (0.03)	3.2 (0.02)	3.2 (0.03)	.72	.89	.89
C-SMILE	3.5 (0.06)	3.3 (0.06)	3.3 (0.06)	.15	.24	.74
Control	3.6 (0.04)	3.5 (0.05)	3.4 (0.04)	.58	.005	.15
$P_{\text{PC-SMILE vs C-SMILE}}$	<.001	.042	.030	*P*-value (interaction)	.60	
$P_{\text{PC-SMILE vs Control}}$	<.001	<.001	.002			
$P_{\text{C-SMILE vs Control}}$	.21	.017	.29			

Table S5. Moderation effects of gender on the treatment effects.

Outcome variables	Baseline	2-week follow-up	3-month follow-up	*P*-value (baseline vs 2-week follow-up)	*P*-value (baseline vs 3-month follow-up)	*P*-value (2-week vs 3-month follow-up)
**RCADS-25: child version-overall**						
Boys						
PC-SMILE	16.1 (2.2)	14.9 (1.9)	15.7 (2.4)	.38	.80	.65
C-SMILE	13.4 (2.1)	14.5 (2.5)	15.6 (2.4)	.65	.28	.51
Control	18.0 (2.5)	15.6 (2.3)	16.5 (2.4)	.27	.39	.68
$P_{\text{PC-SMILE vs C-SMILE}}$	.28	.87	1.0	*P*-value (interaction)	.71	
$P_{\text{PC-SMILE vs Control}}$	.52	.76	.79			
$P_{\text{C-SMILE vs Control}}$	.08	.66	.76			
Girls						
PC-SMILE	19.3 (0.8)	14.8 (0.2)	17.5 (1.0)	<.001	.31	.017
C-SMILE	11.7 (0.5)	9.0 (0.7)	9.9 (0.5)	.012	.052	.36
Control	31.4 (0.5)	29.2 (0.4)	30.7 (0.6)	.003	.45	.09
$P_{\text{PC-SMILE vs C-SMILE}}$	<.001	<.001	<.001	*P*-value (interaction)	.035	
$P_{\text{PC-SMILE vs Control}}$	<.001	<.001	<.001			
$P_{\text{C-SMILE vs Control}}$	<.001	<.001	<.001			
**RCADS-25: child version-anxiety**						
Boys						
PC-SMILE	10.2 (1.4)	9.2 (1.3)	9.5 (1.5)	.26	.54	.80
C-SMILE	8.4 (1.4)	8.5 (1.6)	9.9 (1.5)	.99	.27	.20
Control	11.1 (1.5)	9.8 (1.4)	10.0 (1.5)	.34	.36	.84
$P_{\text{PC-SMILE vs C-SMILE}}$	.27	.63	.82	*P*-value (interaction)	.65	
$P_{\text{PC-SMILE vs Control}}$	.62	.72	.77			
$P_{\text{C-SMILE vs Control}}$	.10	.42	.95			
Girls						
PC-SMILE	12.3 (1.3)	8.7 (1.1)	11.0 (1.4)	<.001	.14	.054
C-SMILE	12.8 (1.2)	11.0 (1.3)	11.2 (1.3)	.040	.028	.81
Control	13.8 (1.3)	12.3 (1.3)	12.7 (1.3)	.045	.17	.63
$P_{\text{PC-SMILE vs C-SMILE}}$	.76	.17	.92	*P*-value (interaction)	.40	
$P_{\text{PC-SMILE vs Control}}$	.34	.012	.30			
$P_{\text{C-SMILE vs Control}}$	.58	.46	.39			
**RCADS-25: child version-depression**						
Boys						

PC-SMILE	5.9 (0.8)	5.7 (0.9)	6.2 (1.0)	.70	.76	.49
C-SMILE	5.0 (0.8)	6.0 (1.0)	5.7 (0.9)	.31	.37	.70
Control	7.0 (1.1)	5.9 (0.9)	6.5 (1.0)	.25	.51	.51
$P_{\text{PC-SMILE vs C-SMILE}}$	.37	.77	.73	*P*-value (interaction)	.60	
$P_{\text{PC-SMILE vs Control}}$	.42	.86	.82			
$P_{\text{C-SMILE vs Control}}$	.09	.90	.54			
Girls						
PC-SMILE	6.6 (0.3)	5.6 (0.2)	6.1 (0.5)	.009	.54	.41
C-SMILE	4.1 (0.2)	3.1 (0.3)	3.7 (0.3)	.006	.33	.31
Control	13.0 (0.1)	12.1 (0.1)	12.8 (0.2)	<.001	.73	.024
$P_{\text{PC-SMILE vs C-SMILE}}$	<.001	<.001	<.001	*P*-value (interaction)	.97	
$P_{\text{PC-SMILE vs Control}}$	<.001	<.001	<.001			
$P_{\text{C-SMILE vs Control}}$	<.001	<.001	<.001			
**Hopelessness**						
Boys						
PC-SMILE	2.3 (0.06)	2.2 (0.08)	2.2 (0.05)	.35	.51	.50
C-SMILE	2.0 (0.09)	2.1 (0.10)	2.0 (0.07)	.53	.80	.51
Control	1.9 (0.07)	2.1 (0.08)	2.1 (0.03)	.23	.011	.87
$P_{\text{PC-SMILE vs C-SMILE}}$	<.001	.39	.026	*P*-value (interaction)	.016	
$P_{\text{PC-SMILE vs Control}}$	<.001	.59	<.001			
$P_{\text{C-SMILE vs Control}}$	.69	.81	.21			
Girls						
PC-SMILE	2.2 (0.2)	2.0 (0.2)	2.1 (0.1)	.018	.33	.13
C-SMILE	2.2 (0.2)	2.1 (0.2)	2.2 (0.2)	.44	.87	.47
Control	2.3 (0.2)	2.3 (0.2)	2.2 (0.2)	.81	.28	.37
$P_{\text{PC-SMILE vs C-SMILE}}$	.85	.49	.83	*P*-value (interaction)	.38	
$P_{\text{PC-SMILE vs Control}}$	.49	.036	.56			
$P_{\text{C-SMILE vs Control}}$	.44	.28	.77			
**Mental well-being**						
Boys						
PC-SMILE	23.7 (0.8)	24.5 (1.0)	24.2 (1.0)	.41	.62	.84
C-SMILE	25.4 (0.9)	23.8 (1.0)	23.5 (1.0)	.08	.013	.74
Control	22.9 (0.9)	23.8 (1.0)	22.7 (1.0)	.43	.82	.33
$P_{\text{PC-SMILE vs C-SMILE}}$	.13	.66	.60	*P*-value (interaction)	.14	
$P_{\text{PC-SMILE vs Control}}$	.50	.62	.25			
$P_{\text{C-SMILE vs Control}}$	.043	.95	.55			
Girls						
PC-SMILE	25.2 (0.4)	26.0 (0.5)	25.6 (0.4)	.35	.55	.53
C-SMILE	25.4 (0.1)	26.2 (0.3)	25.7 (0.3)	.008	.34	.36

Control	19.6 (0.3)	20.2 (0.2)	20.0 (0.2)	.22	.36	.66
$P_{\text{PC-SMILE vs C-SMILE}}$	.67	.47	.38	*P*-value (interaction)	.94	
$P_{\text{PC-SMILE vs Control}}$	<.001	<.001	<.001			
$P_{\text{C-SMILE vs Control}}$	<.001	<.001	<.001			
**Parent–child interactions**						
Boys						
PC-SMILE	2.7 (0.3)	2.7 (0.3)	2.8 (0.3)	.79	.58	.79
C-SMILE	2.6 (0.3)	2.6 (0.3)	2.6 (0.3)	.77	.95	.80
Control	3.1 (0.3)	3.0 (0.3)	3.1 (0.3)	.51	.76	.69
$P_{\text{PC-SMILE vs C-SMILE}}$	.92	.64	.64	*P*-value (interaction)	.96	
$P_{\text{PC-SMILE vs Control}}$	.16	.54	.47			
$P_{\text{C-SMILE vs Control}}$	.17	.35	.27			
Girls						
PC-SMILE	3.3 (0.3)	3.3 (0.3)	3.4 (0.3)	.96	.62	.69
C-SMILE	3.4 (0.3)	3.4 (0.3)	3.1 (0.3)	.90	.14	.09
Control	3.2 (0.3)	3.3 (0.3)	3.1 (0.3)	.43	.64	.20
$P_{\text{PC-SMILE vs C-SMILE}}$	.80	.77	.47	*P*-value (interaction)	.60	
$P_{\text{PC-SMILE vs Control}}$	.77	.94	.40			
$P_{\text{C-SMILE vs Control}}$	.61	.84	.92			
**Parent–child relationships**						
Boys						
PC-SMILE	2.8 (0.1)	2.9 (0.1)	2.9 (0.1)	.19	.031	.66
C-SMILE	2.6 (0.1)	2.7 (0.1)	2.6 (0.1)	.74	.98	.75
Control	2.8 (0.1)	3.0 (0.1)	2.9 (0.1)	.16	.57	.46
$P_{\text{PC-SMILE vs C-SMILE}}$	.38	.15	.07	*P*-value (interaction)	.77	
$P_{\text{PC-SMILE vs Control}}$	.76	.55	.80			
$P_{\text{C-SMILE vs Control}}$	.26	.049	.14			
Girls						
PC-SMILE	2.8 (0.1)	3.0 (0.1)	3.0 (0.1)	.017	.037	.60
C-SMILE	2.9 (0.1)	2.9 (0.1)	2.9 (0.1)	.72	.92	.75
Control	2.9 (0.1)	2.9 (0.1)	2.8 (0.1)	.93	.12	.12
$P_{\text{PC-SMILE vs C-SMILE}}$	.71	.46	.54	*P*-value (interaction)	.11	
$P_{\text{PC-SMILE vs Control}}$	.75	.37	.15			
$P_{\text{C-SMILE vs Control}}$	.98	.86	.38			
**Academic self-efficacy**						
Boys						

PC-SMILE	3.8 (0.1)	3.8 (0.1)	4.0 (0.1)	.89	.34	.30
C-SMILE	3.8 (0.2)	3.7 (0.2)	3.4 (0.2)	.54	.027	.22
Control	3.9 (0.2)	3.9 (0.2)	4.0 (0.2)	.91	.26	.38
$P_{\text{PC-SMILE vs C-SMILE}}$	.92	.51	.022	*P*-value (interaction)	.13	
$P_{\text{PC-SMILE vs Control}}$	.79	.80	.79			
$P_{\text{C-SMILE vs Control}}$	.73	.43	.023			
Girls						
PC-SMILE	4.4 (0.1)	4.3 (0.1)	4.3 (0.1)	.47	.28	.70
C-SMILE	4.2 (0.1)	4.4 (0.1)	4.4 (0.2)	.18	.20	.94
Control	4.2 (0.1)	4.2 (0.1)	4.2 (0.2)	.43	.62	.87
$P_{\text{PC-SMILE vs C-SMILE}}$	.35	.80	.63	*P*-value (interaction)	.41	
$P_{\text{PC-SMILE vs Control}}$	.39	.37	.67			
$P_{\text{C-SMILE vs Control}}$	.90	.30	.43			
**Perceived parent performance (vs. learning) orientation**						
Boys						
PC-SMILE	3.5 (0.1)	3.3 (0.1)	3.4 (0.1)	.18	.31	.65
C-SMILE	3.5 (0.1)	3.4 (0.1)	3.4 (0.1)	.23	.031	.26
Control	3.6 (0.1)	3.4 (0.1)	3.4 (0.1)	.036	.09	.70
$P_{\text{PC-SMILE vs C-SMILE}}$	.56	.39	.59	*P*-value (interaction)	.78	
$P_{\text{PC-SMILE vs Control}}$	.44	.60	.72			
$P_{\text{C-SMILE vs Control}}$	.89	.71	.41			
Girls						
PC-SMILE	3.33 (0.1)	3.36 (0.1)	3.30 (0.1)	.74	.66	.46
C-SMILE	3.40 (0.1)	3.22 (0.1)	3.35 (0.1)	.024	.55	.033
Control	3.50 (0.1)	3.49 (0.1)	3.35 (0.1)	.85	.036	.07
$P_{\text{PC-SMILE vs C-SMILE}}$	.59	.24	.68	*P*-value (interaction)	.042	
$P_{\text{PC-SMILE vs Control}}$	.16	.29	.68			
$P_{\text{C-SMILE vs Control}}$	.40	.036	1.0			
